# Using *Plasmodium knowlesi* as a model for screening *Plasmodium vivax* blood-stage malaria vaccine targets reveals new candidates

**DOI:** 10.1371/journal.ppat.1008864

**Published:** 2021-07-01

**Authors:** Duncan N. Ndegwa, Prasun Kundu, Jessica B. Hostetler, Alejandro Marin-Menendez, Theo Sanderson, Kioko Mwikali, Lisa H. Verzier, Rachael Coyle, Sophie Adjalley, Julian C. Rayner

**Affiliations:** 1 Wellcome Sanger Institute, Wellcome Genome Campus, Hinxton, Cambridge, United Kingdom; 2 Department of Biological Sciences, University of Embu, Embu, Kenya; 3 Cambridge Institute for Medical Research, University of Cambridge, Cambridge Biomedical Campus, Hills Road, Cambridge, United Kingdom; Burnet Institute, AUSTRALIA

## Abstract

*Plasmodium vivax* is responsible for the majority of malaria cases outside Africa. Unlike *P*. *falciparum*, the *P*. *vivax* life-cycle includes a dormant liver stage, the hypnozoite, which can cause infection in the absence of mosquito transmission. An effective vaccine against *P*. *vivax* blood stages would limit symptoms and pathology from such recurrent infections, and therefore could play a critical role in the control of this species. Vaccine development in *P*. *vivax*, however, lags considerably behind *P*. *falciparum*, which has many identified targets with several having transitioned to Phase II testing. By contrast only one *P*. *vivax* blood-stage vaccine candidate based on the Duffy Binding Protein (PvDBP), has reached Phase Ia, in large part because the lack of a continuous *in vitro* culture system for *P*. *vivax* limits systematic screening of new candidates. We used the close phylogenetic relationship between *P*. *vivax* and *P*. *knowlesi*, for which an *in vitro* culture system in human erythrocytes exists, to test the scalability of systematic reverse vaccinology to identify and prioritise *P*. *vivax* blood-stage targets. A panel of *P*. *vivax* proteins predicted to function in erythrocyte invasion were expressed as full-length recombinant ectodomains in a mammalian expression system. Eight of these antigens were used to generate polyclonal antibodies, which were screened for their ability to recognize orthologous proteins in *P*. *knowlesi*. These antibodies were then tested for inhibition of growth and invasion of both wild type *P*. *knowlesi* and chimeric *P*. *knowlesi* lines modified using CRISPR/Cas9 to exchange *P*. *knowlesi* genes with their *P*. *vivax* orthologues. Candidates that induced antibodies that inhibited invasion to a similar level as PvDBP were identified, confirming the utility of *P*. *knowlesi* as a model for *P*. *vivax* vaccine development and prioritizing antigens for further follow up.

## Author summary

Malaria parasites cause disease after invading human red blood cells, implying that a vaccine that interrupts this process could play a significant role in malaria control. Multiple *Plasmodium* parasite species can cause malaria in humans, and most malaria outside Africa is caused by *Plasmodium vivax*. There is currently no effective vaccine against the blood stage of any malaria parasite, and progress in *P*. *vivax* vaccine development has been particularly hampered because this parasite species cannot be cultured for prolonged periods of time in the lab. We explored whether a related species, *P*. *knowlesi*, which can be propagated in human red blood cells *in vitro*, can be used to screen for potential *P*. *vivax* vaccine targets. We raised antibodies against selected *P*. *vivax* proteins and tested their ability to recognize and prevent *P*. *knowlesi* parasites from invading human red blood cells, thereby identifying multiple novel vaccine candidates.

## Introduction

Malaria remains a major global health challenge, with an estimated 228 million cases and >400,000 deaths in 2018 [1]. While there are five *Plasmodium* species that can cause malaria in humans, the majority of clinical cases are caused by *P*. *falciparum* and *P*. *vivax*. *P*. *falciparum* causes almost all malaria cases in Africa, but *P*. *vivax* is the dominant cause of malaria in the Americas, and causes a similar number of cases as *P*. *falciparum* in South-east Asia [1]. As well as having different global distributions, the two species are also very different biologically, which has significant implications for control. *P*. *vivax*, along with *P*. *ovale*, can form hypnozoites during its liver stage, which are quiescent forms of the parasite that remain dormant from weeks to years in the liver, re-emerging upon stimulation to cause a relapse of malaria. Hypnozoites can therefore act as a continuous source of infection even in the absence of active transmission. This hurdle is made more significant by the fact that primaquine and tafenoquine, the only drugs used to treat hypnozoites, are frequently contraindicated due to their toxicity in patients with glucose-6-phosphate deficiency, a common polymorphism in regions of the world where *P*. *vivax* is most prevalent [2]⁠. In addition, sexual stage development in *P*. *vivax* is much more rapid than in *P*. *falciparum* [3], meaning that even with rapid treatment with antimalarials, onwards transmission can still occur. These features limit the effectiveness of current chemotherapeutic interventions, making the search for an effective vaccine even more important for *P*. *vivax*.

The complex life-cycle of *Plasmodium* parasites present multiple potential intervention strategies, including preventing transmission to the mosquito, targeting the liver stage to prevent disease and relapse, and targeting blood stages to limit disease and also potentially lower the potential of transmission from one infected individual to another. Vaccine targets across all these stages of the parasite are under investigation, although in general far fewer antigens have been studied in depth in *P*. *vivax* than in *P*. *falciparum* (reviewed in [4–6]). This is particularly the case for blood stage targets, where only a few targets such as *P*. *vivax* apical membrane antigen 1 (PvAMA1) [7,8] and *P*. *vivax* merozoite surface protein 1 (PvMSP1_19_) [9,10] have advanced to pre-clinical study. The furthest advanced *P*. *vivax* vaccines, by far, are based on *P*. *vivax* Duffy Binding Protein (PvDBP), the only blood stage target that has reached clinical Phase Ia trials [11–15]⁠⁠⁠. This is in stark contrast to *P*. *falciparum*, where multiple targets in different stages have been tested in Phase Ia (reviewed in [4]), and the RTS, S pre-erythrocytic vaccine has advanced beyond Phase III to pilot testing across three countries in Africa [16]. More *P*. *vivax* targets clearly need to be screened if vaccine development for this species is to advance.

It was previously believed that *P*. *vivax was* completely dependent on the interaction between PvDBP and its receptor, Duffy Antigen Receptor for Chemokine (DARC) [17–20] to invade human erythrocytes. However, it has recently been shown that *P*. *vivax* is also able to infect individuals who are Duffy negative, so express little or no DARC on the surface of their erythrocytes [21–23]. While the invasion of Duffy negative erythrocytes could still rely on PvDBP [24,25], the sole focus on PvDBP as a vaccine candidate clearly needs to be reassessed and additional targets evaluated, either as potential substitutes for PvDBP, or to be used in combination with it. As noted above, erythrocyte invasion is a very complex process, and while the process is much less well-understood in *P*. *vivax* than it is in *P*. *falciparum* [26]⁠, multiple other *P*. *vivax* ligands such as reticulocyte-binding protein 2 (RBP2b) [27], GPI-anchored micronemal antigen (GAMA) [28]⁠, and erythrocyte binding protein 2 (ebp2) [29] have all been shown or proposed to be involved in invasion, and could potentially be used alone or in combination with PvDBP to target multiple steps of invasion, an approach that shows some preliminary promise in *P*. *falciparum* [30]⁠.

In this study we took a reverse vaccinology approach to identify new *P*. *vivax* vaccine targets, building on previous work where we expressed a panel of 37 full-length recombinant *P*. *vivax* vaccine targets predicted to be involved in erythrocyte invasion [31]. Polyclonal antibodies were generated against 8 of these proteins, and the antibodies were tested for their ability to inhibit merozoite invasion. *P*. *vivax* preferentially invades immature erythrocytes [32] making even short-term culture of *P*. *vivax* exceedingly difficult [33–36], and limiting the development of continuous culture of *P*. *vivax in vitro*, despite herculean efforts [37]. As a first-stage screen we therefore performed invasion inhibition assays using *P*. *knowlesi*, a close phylogenetic relative of *P*. *vivax* [38,39] that has been adapted to *in vitro* culture in human erythrocytes [40,41] and has been used previously as a model to assess *P*. *vivax* blood-stage vaccine candidates [42,43]. *P*. *knowlesi* is highly amenable to genetic manipulation [40] and has been used in several experimental genetic studies to investigate the role of specific genes in erythrocyte invasion [44,45]. The adaptation of CRISPR-Cas9 genomic modification to *P*. *knowlesi* has expanded the utility of this model further, by facilitating the direct swap of *P*. *knowlesi* genes for their *P*. *vivax* counterparts [46], an approach that has already been used to aid the further development of PvDBP as a vaccine candidate [47]. We combined screening against wild-type and genome modified *P*. *knowlesi* parasites to prioritise new targets for *P*. *vivax* blood-stage vaccine development, and in so doing present further evidence that *P*. *knowlesi* can be used as a readily manipulatable *in vitro* model for *P*. *vivax*.

## Results

### Generation of polyclonal antibodies against new *P*. *vivax* vaccine candidates

We have previously expressed a pilot library of 37 *P*. *vivax* proteins that were either shown to localise to merozoite organelles with a role in invasion, or were predicted to do so based on the localisation of their respective *P*. *falciparum* homologues [31]. In all cases, the full-length extracellular domains of these proteins were expressed using a mammalian protein expression system. This approach, which increases the likelihood of correct folding of disulphide-linked extracellular domains, has been used extensively for *P*. *falciparum* invasion-associated proteins [48] to generate antigens capable of inducing potent invasion-inhibitory antibodies [30], including for the major *P*. *falciparum* blood-stage vaccine target PfRh5 [49]⁠. Comparing immunoreactivity of native or heat-denatured epitopes and testing for protein-protein interactions indicated that the previously produced *P*. *vivax* library was likely to consist of largely functional proteins [31]⁠. To test whether this library could also be used to generate inhibitory antibodies, rabbit polyclonal antibodies were raised against eight targets, selected to represent a range of predicted subcellular localizations and including PvDBP as a positive control (Table 1). In all cases, antibodies were raised against the complete recombinant ectodomain of the candidates. The immunisation schedule is outlined in the Methods, and total IgG was purified from serum using Protein A affinity chromatography to use in downstream assays.

**Table 1:** *Plasmodium vivax* vaccine candidates.

*P*. *vivax* targets accession number	Protein product	Abbreviation	*P*. *knowlesi* orthologue	Protein pairwise identify between Pv and Pk
PVX_088910	GPI-anchored micronemal antigen	PvGAMA	PKNH_1332900 (PkGAMA)	66%
PVX_113775	6-cysteine protein p12	PvP12	PKNH_1137300 (PkP12)	70%
PVX_097720	Merozoite Surface Protein 3	PvMSP3.10 PvMSP3H PvMSP3alpha	PKNH_1030500 (PkMSP3)	9%
PVX_082700	Merozoite Surface Protein 7	PvMSP7.1	PKNH_1265900 PKNH_1266000 PKNH_1266100 PKNH_1266300 (PkMSP7 like proteins)	16% 23% 18% 21%
PVX_090210	Asparagine-rich protein	PvARP	PKNH_0515300 (PkARP)	56%
PVX_09240	Cysteine-rich Protective Antigen	PvCyRPA	PKNH_0515800 (PkCyRPA)	68%
PVX_110810	Duffy Binding Protein	PvDBP	PKNH_0623500 (PkDBPalpha)	51%
PVX_000995	6-cysteine protein p41	PvP41	PKNH_0303000 (PkP41)	80%

### Anti-*Plasmodium vivax* antibodies are able to recognise orthologues in *P*. *knowlesi*

Given the difficulty in acquiring *P*. *vivax ex vivo* isolates for testing and technical issues with even short-term culture, we used *P*. *knowlesi* as a model for screening new blood-stage vaccine candidates, an approach that has previously been pioneered using both wildtype and genetically modified parasite lines [42,43,46,47]. *P*. *knowlesi* naturally infects the Kra cynomolgus macaque (*Macaca fascicularis*) but causes severe zoonotic malaria in Southeast Asia [50], and critically falls into the same clade of parasites as *P*. *vivax* [39]. While this phylogenetic relationship is reflected in an overall higher degree of conservation between the *P*. *vivax* and *P*. *knowlesi* genomes than the *P*. *vivax* and *P*. *falciparum* genomes [39], the degree of conservation varies at the individual gene level. Sequence alignment between our *P*. *vivax* candidate proteins and their orthologues in *P*. *knowlesi* showed a range of sequence similarities (Table 1), from a pairwise identity score of 51% for PvDBP and its closest *P*. *knowlesi* orthologue PkDBPα, to higher identity scores for several targets (*P*. *vivax* GPI-anchored micronemal protein; PvGAMA, *P*. *vivax* 6-cysteine protein P12 (Pv12); *P*. *vivax* Asparagine-rich Protein (PvARP); *P*. *vivax* Cysteine-rich Protective Antigen (PvCyRPA); *P*. *vivax* 6-cysteine protein P41 (Pv41)), reaching 80% in the case of Pv41. In contrast the *P*. *vivax* Merozoite Surface Proteins 3.10 (PvMSP3.10) and *P*. *vivax* Merozoite Surface Proteins 7.1 (PvMSP7.1), both members of multigene families which are known to be highly polymorphic within and between *Plasmodium* species, were chosen to represent proteins with a lower degree of conservation.

**Fig 1:**
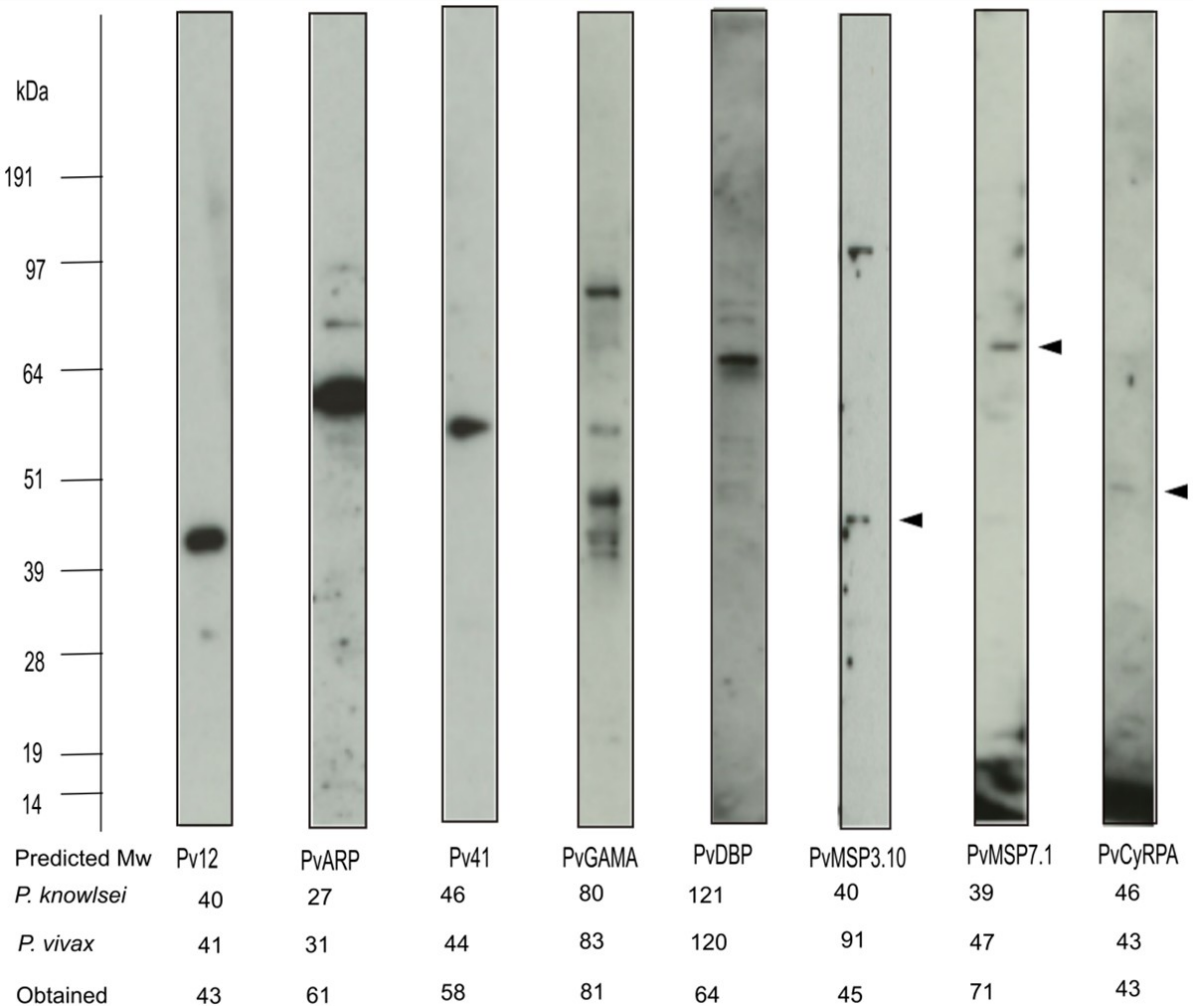
Anti-*P*. *vivax* polyclonal antibodies recognise proteins in *P*. *knowlesi* schizont protein lysates. Protein extracts from enriched schizont-stage *P*. *knowlesi* cultures were separated on SDS-PAGE gels, transferred to nitrocellulose and probed with anti-*P*. *vivax* polyclonal antibodies. Obtained size corresponding to the major bands seen with each antibody is indicated, along with the expected size of the corresponding protein in *P*. *knowlesi* (upper row) and its orthologue in *P*. *vivax* (middle row) in kiloDaltons (kDa). The arrows indicate the major bands for PvMSP3.10, PVMSP7.1 and PvCyRPA. Note that the expected size of PkMSP7.1 of 39kDa is an average of the molecular weight of four PkMSP7 like proteins (i.e. PKNH_1265900, PKNH_1266000, PKNH_1266100, PKNH_1266300) that range from 32–46 kDa.

To explore whether variable degrees of homology would limit our ability to test specific targets in *P*. *knowlesi*, we first determined whether antibodies raised against *P*. *vivax* (Pv) targets can recognise their *P*. *knowlesi* (Pk) orthologues in immunoblots using *P*. *knowlesi* schizont-stage protein lysates (Fig 1). Antibodies raised against Pv12, PvARP, Pv41, PvMSP7.1 and PvDBP produced a single immunoreactive band, while several bands were detected with antibodies against PvGAMA, PvMSP3.10 and PvCyRPA, suggesting either post-translational modifications or proteolytic processing events, as is known to frequently occur in *Plasmodium* merozoite proteins both *in vitro* and when expressed recombinantly in heterologous systems [48]. While multiple factors could affect signal strength, including expression level in schizont stages, there was some correlation between % Pv/Pk identity and the strength of the immunoblot signal, PvCyRPA being the exception with a weak detection signal despite 68% identity. Antibodies against Pv12, PvGAMA, PvMSP3.10, PvCyRPA all detected proteins around their expected molecular weight based on estimates from the corresponding orthologous protein in *P*. *knowlesi*. In contrast, anti-PvARP, Pv41 and PvMSP7.1 detected proteins larger than the expected molecular weight suggesting that they might migrate more slowly, which again is not uncommon in extracellular *Plasmodium* proteins. Anti-PvDBP detected a protein half the expected size suggesting either that PkDBPα (the closest *P*. *knowlesi* orthologue of PvDBP) is highly processed, or that anti-PvDBP antibodies cross-react with multiple PkDBP proteins. Overall however, immunoblotting showed that the majority of anti-*P*. *vivax* antibodies recognised *P*. *knowlesi* proteins.

### Anti-*P*. *vivax* antibodies are able to localise orthologous target proteins to *P*. *knowlesi* invasion organelles

**Fig 2:**
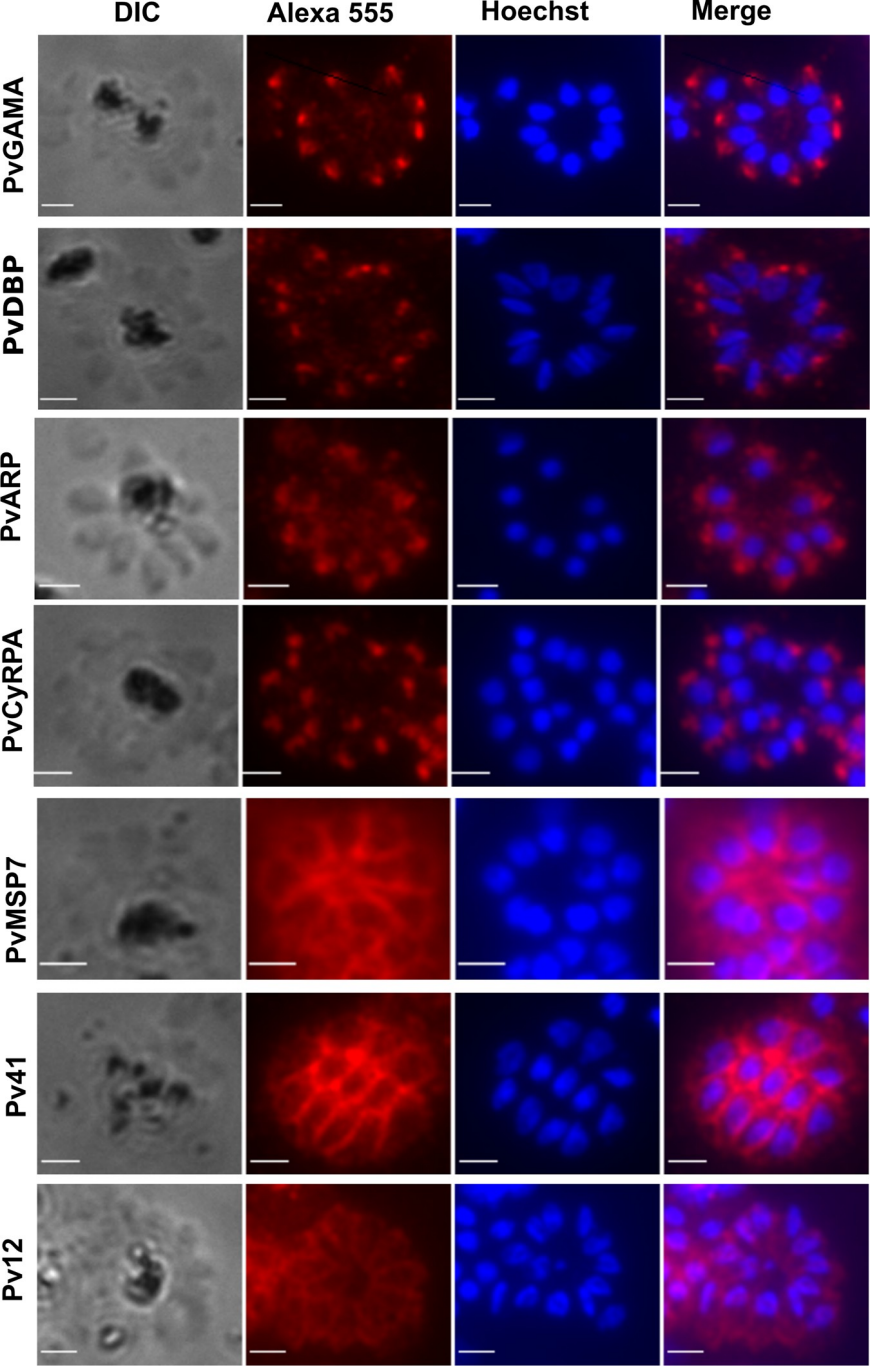
Immunolocalisation of *P*. *knowlesi* proteins using polyclonal anti-*P*. *vivax* antibodies. Antibodies raised against *P*. *vivax* vaccine candidates were used in immunofluorescence assays using enriched schizont-stage *P*. *knowlesi* parasites. Rabbit polyclonals were detected using Alexa 555-labeled anti-rabbit secondary antibody, and compared with parasite nuclei as detected with Hoechst 33342. Merge is an overlay of Alexa 555 and Hoeschst. Scale bar is 2 micrometers.

To further explore the specificity of our anti-*P*. *vivax* antibody panel, we used them in indirect immunofluorescence assays to test their ability to recognise mature *P*. *knowlesi* schizonts (Fig 2). Out of the 8 polyclonals, only anti-PvMSP3.10 (which shows the lowest percentage of identity between Pv and Pk) did not produce a specifically localised signal (Fig 2). Anti-PvGAMA, PvCyRPA, PvDBP and PvARP all labelled punctate foci within the merozoites, while anti-Pv12, Pv41 and PvMSP7.1 all appeared to label the entire merozoite surface. No staining was observed with pre-immune antiserum (S1 Fig), confirming that the labelling was antigen-specific.

**Fig 3:**
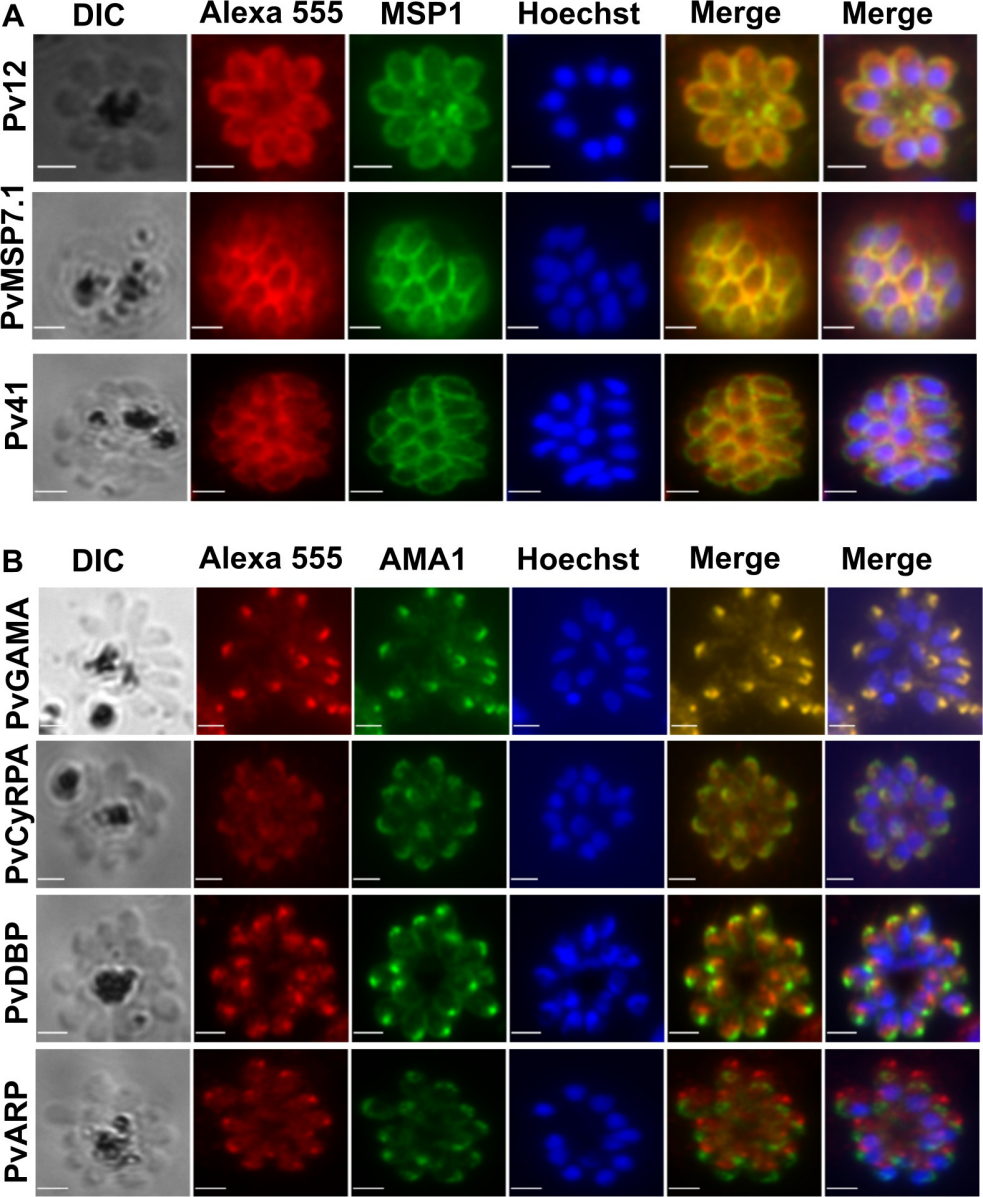
Colocalisation of *P*. *knowlesi* homologs of *P*. *vivax* vaccine candidates with antibodies to proteins of known cellular location. *P*. *knowlesi* proteins were localised using rabbit polyclonal antibodies raised against *P*. *vivax* vaccine candidates and co-stained with rat antibodies against A) anti-PkMSP1 or B) anti-PfAMA1 for colocalization (origin of antibodies is described in Methods). Alexa Fluor 555 goat-anti rabbit and Alexa Fluor 488 goat-anti rat were used as secondary antibodies, and parasite DNA localised using Hoechst 33342. The first Merge column is an overlay of Alexa 555 and Alexa488 staining, while the second Merge column is an overlay of Alexa 555, Alexa488 and Hoeschst. Scale bar is 2 micrometers.

To establish the specific location of each antigen, anti-*P*. *vivax* antibodies were used in co-localization experiments with antibodies specific to proteins of known cellular locations: AMA1, MSP1-19 and GAP45 which are located in microneme, merozoite surface and inner membrane complex (IMC), respectively (see Methods for antibody sources). Anti-Pv12, Pv41 and PvMSP7.1 all showed a clear co-localization with anti-MSP1 (Figs 3A and S2A) suggesting that their orthologous targets are located on the merozoite surface. Anti-PvGAMA and, to a lesser extent, anti-PvCyRPA and anti-PvDBP, co-localised with anti-AMA1, suggesting that their orthologous targets are located in apical secretory organelles such as the micronemes (Figs 3B and S3). Anti-ARP appeared to be apically located but did not co-localise with any markers that we tested (Figs 3B and S3), such that its exact location remains to be determined. To confirm that antibodies against Pv12, Pv41 and PvMSP7.1 were labelling the merozoite surface and not the IMC, markers of which produce similar staining patterns in late schizonts, co-staining with the IMC marker anti-GAP45 was also carried out in late trophozoites/early schizonts, as the IMC and merozoite surface are easier to distinguish earlier in the cell cycle. In all cases there was no co-localisation with anti-GAP45 in these earlier stages (S2B Fig), confirming a merozoite surface, not an IMC, location.

### Screening anti-*P*.*vivax* antibodies for inhibitory activity in *P*. *knowlesi* identifies novel invasion-blocking candidates

**Fig 4:**
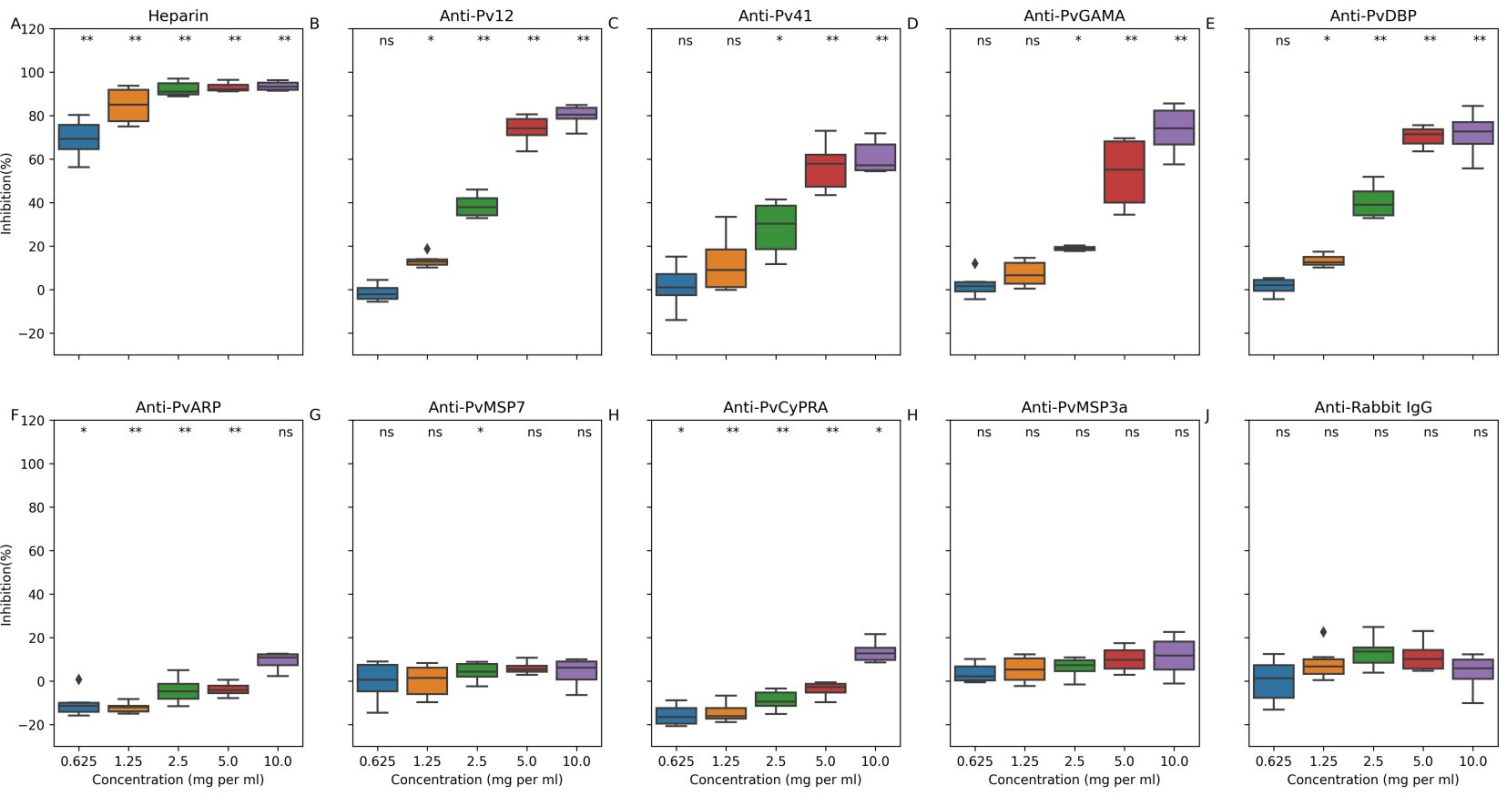
Dose-dependent inhibition of wild-type *P*. *knowlesi* by polyclonal antibodies to *P*. *vivax* vaccine candidates. *P*. *knowlesi* parasites were grown in the presence of a dilution series of anti-*P*. *vivax* antibodies, with invasion events into fluorescently labelled red blood cells quantified using dual colour flow cytomtery (method summarised in S4 Fig). The effect of antibodies was calculated as a percentage, comparing invasion in the presence of antibodies to invasion in the absence of antibodies. Heparin and total IgG from unimmunised rabbits were used as positive and negative controls respectively. The data presented includes observations from two independent experiments, each of which had three replicate wells for each condition/antibody concentration. To determine if the inhibition was statistically significant, all treatments were independently compared to the effect of the same concentration of non-immune rabbit IgG using Mann-Whitney-Wilcoxon test: *p = 0.05–0.01; **p<0.01.

Having established that anti-*P*. *vivax* antibodies could be used to specifically detect homologues in *P*. *knowlesi*, we explored whether the same antibodies could inhibit *P*. *knowlesi* erythrocyte invasion. Serial two-fold dilutions of purified total IgG were prepared starting from 10 mg/ml, and incubated with synchronized ring-stage *P*. *knowlesi* parasites for 24 hours in the presence of CellTrace Far Red uninfected erythrocytes. Dual colour flow cytometry was used to measure invasion, which was quantified as the percentage of erythrocytes that were both SYBR green and CellTrace Far Red positive after 24 hours, compared to assays performed in the absence of any antibodies or in the presence of pre-immune IgG (see S4 Fig). All antibody combinations were carried out in two biological replicates, each consisting of three technical replicates. As shown in Fig 4, compared to the positive and negative controls for inhibition (heparin and rabbit IgG respectively), antibody activity fell into two broad groups: inhibitory (Fig 4B–4E, anti-Pv12, Pv41, PvGAMA and PvDBP which gave IC_50_ values of 4.5, 5.6, 6.1 and 4.2 mg/ml respectively) and not inhibitory (Fig 4F–4J, anti-PvARP, PvCyRPA, PvMSP7.1, PvMSP3.10). The low level of inhibition observed with anti-PvMSP7.1 and PvMSP3.10 could be due to the low degree of homology between Pv and Pk homologs, and the lack of inhibition observed with the anti-PvMSP3.10 was consistent with the absence of cross-reactivity with PkMSP3 homologues in immunofluorescence assays. Strong inhibition with anti-PvDBP, the only *P*. *vivax* blood stage vaccine target in the advanced stage of vaccine development, was confirmatory and comparable to other studies [46]⁠. Antibodies to two other targets, Pv12 and PvGAMA, had similar IC_50_s to anti-PvDBP, while antibodies to Pv41, which interacts with Pv12 [31], also had strong inhibition. The levels of total IgG that was required to inhibit invasion was broadly similar to that observed in other studies of *P*. *falciparum* vaccine targets [30], including the major target PfRH5 [49,51] when total IgG is being used in such assays. Purifying antigen-specific antibodies from polyclonal serum would likely have reduced the concentration required for inhibition just as it does for PfRH5 in both human and non-human primate vaccination studies [52,53], but was not possible in this study because of the number of antibodies and assays being carried out.

### Gene editing in *P*. *knowlesi* establishes that Pk41 and PkGAMA are not essential for blood-stage growth

Gene essentiality is one potential prioritisation factor in ranking vaccine candidates, as targeting the product of a gene that is absolutely required for parasite development is by definition more likely to yield growth inhibitory activity. Given that antibodies against Pv12, Pv41, and PvGAMA all inhibited *P*. *knowlesi* growth, we used genome editing to determine whether the orthologous genes in *P*. *knowlesi* could be knocked out. We also targeted *PkARP* as a positive control, as anti-PvARP antibodies had no inhibitory effects on *P*. *knowlesi* (Fig 4), while constructs targeting *PkDBPα* were included as a negative control, as this gene has previously been shown to be essential for invasion of human erythrocytes [46,54]⁠. Gene targeting was carried out using a CRISPR-Cas9 two-vector approach, with one vector containing Cas9 and guide RNA expression cassettes as well as the selection marker, while the other one was a donor template for repair consisting of eGFP flanked by 5’ and 3’ untranslated regions of each respective gene; three different gRNA constructs were generated and used for every gene and successful knockout resulted in the replacement of the endogenous gene with a GFP expression cassette (S5A Fig). Transfection of *P*. *knowlesi* was followed by selection with 100 nM pyrimethamine for 6 days to select for Cas9 expression, and cultures were maintained for up to 3 weeks. Transfections were repeated at least twice for each pair of constructs.

Parasites were recovered from all transfections. Genomic DNA was extracted from recovered lines, and used for genotyping to establish whether integration had occurred. Only parasites transfected with *Pk41* and *PkGAMA* knockout constructs gave bands of the size expected if gene deletion had occurred (S5B Fig). Whole genome sequencing analysis confirmed this result, showing no reads mapping to the deleted regions of the wildtype (WT) *P*. *knowlesi* genome (S6 and S7 Figs), indicating that integration of the knock-out construct had occurred. By contrast, genotyping of parasites transfected with *Pk12*, *PkARP* and *PkDBPα* constructs did not differ from WT cultures. No WT band was amplified for *Pk41* and *PkGAMA* knockout lines, whereas WT parasite controls yielded bands of the expected size (S5B Fig). *Pk41* and *PkGAMA* therefore appear to be non-essential for *P*. *knowlesi* growth, whereas *Pk12*, *PkARP* and *PkDBPα* were not able to be disrupted using this approach.

**Fig 5:**
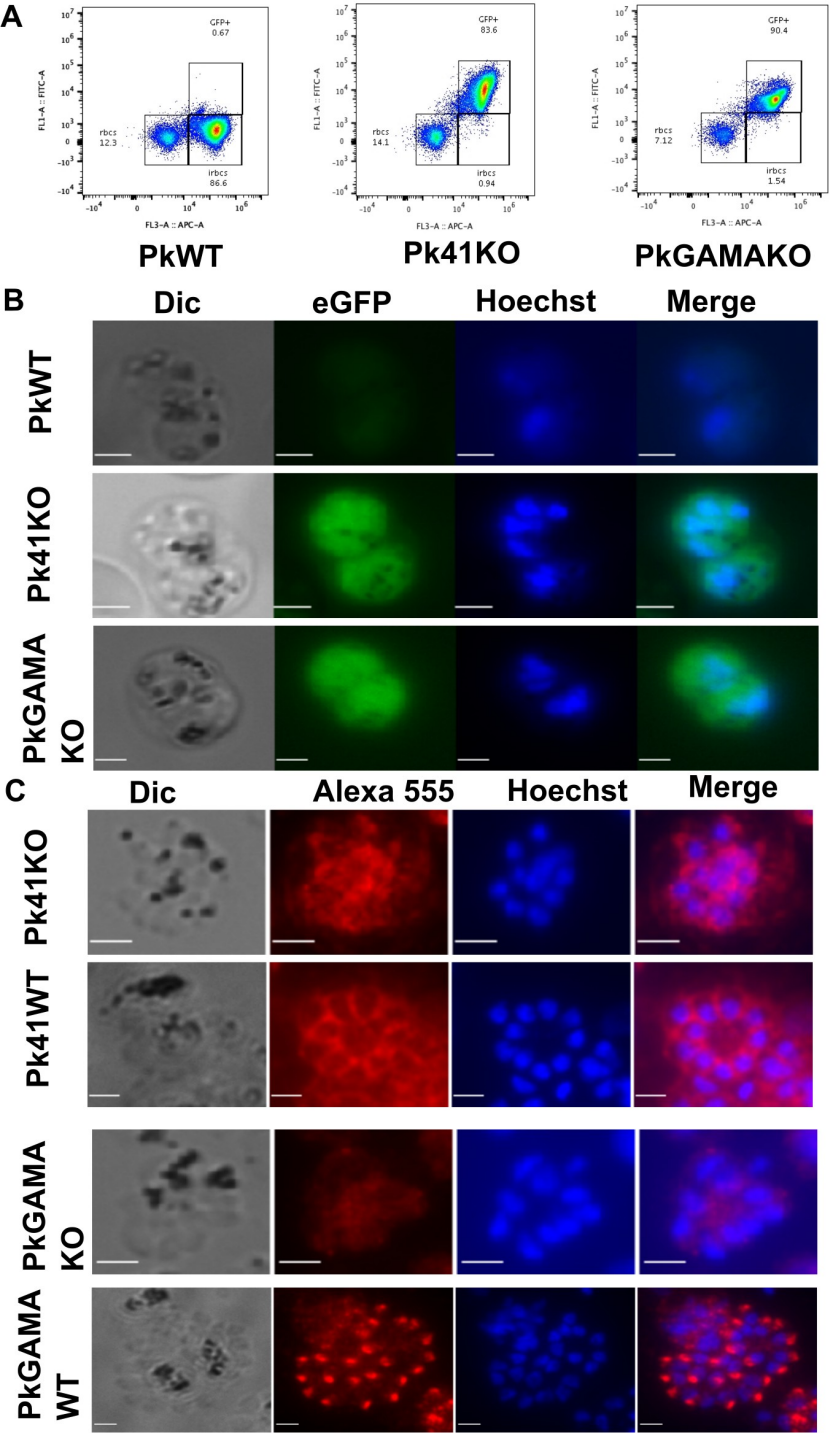
Gene editing can delete PkP41 and PkGAMA in *P*. *knowlesi*. Pkp41 and PkGAMA were replaced with eGFP to generate Pk41KO and PkGAMAKO strains respectively, as described in the Methods. A) Flow cytometry to establish eGFP expression in knockout lines. Enriched knock out and wild-type *P*. *knowlesi* cultures at late stages were labelled with SYBR green in 1X PBS and incubated for one hour after which they were quantified by flow cytometry. Events were gated as both SYBR green and GFP negative (lower left—uninfected RBCs), SYBR green only positive (lower right—RBCs infected with parasites not expressing GFP) and both SYBR green and GFP positive (upper right—RBCs infected with parasites not expressing GFP). Both KO lines expressed GFP, confirming integration of the KO cassette. B) eGFP expression in knock-out *P*. *knowlesi* strains as compared to wild-type *P*. *knowlesi*, imaged using fluorescence microscopy. Parasite nuclei were stained using Hoechst 33342; Merge is an overlay of eGFP and Hoeschst, confirming parasite expression of GFP. Scale bar is 2 micrometers. C) Proteins in knock out and wild-type *P*. *knowlesi* strains were localised using *P*. *vivax* polyclonal antibodies and Alexa Fluor 555 labeled secondary antibody then imaged using fluorescence microscopy. Localisation in both knock out and wild-type Pv41 and PvGAMA was performed using anti-Pv41 and anti-PvGAMA antibodies respectively; in both cases the KO line produced only diffuse background staining, confirming knockout of the *P*. *knowlesi* genes. Parasite nuclei were stained with Hoechst 33342; Merge is an overlay of Alexa 555 and Hoeschst. Scale bar is 2 micrometers.

To confirm that Pk41 and PkGAMA expression was absent in the knockout lines, fluorescence and immunofluorescence assays were performed. Both knockout lines expressed eGFP (Fig 5A and 5B), while localisation assays with anti-Pv41 and anti-PvGAMA gave no specific signal (Fig 5C), showing only background staining in clear contrast to WT parasites (Figs 3 and 4). This confirms that these parasites were not expressing Pk41 and PkGAMA, and therefore that *Pk41* and *PkGAMA* are redundant for intra-erythrocytic growth, despite the fact that anti-P41 and anti-GAMA antibodies were shown to inhibit parasite growth (Fig 4). Growing the knockout strains over nineteen cycles showed both knockout strains grew slightly, but significantly, slower than wild-type strains (S8 Fig; Gamako vs WT; p41ko vs WT), suggesting that while these genes play some role in *in vitro* growth, even if they are not absolutely required for it.

### Allele replacement of *P*. *knowlesi* genes with *P*. *vivax* orthologues increases the inhibitory effect of anti-*P*. *vivax* antibodies

A true test of the inhibitory effectiveness of the anti-*P*. *vivax* antibodies would be in the context of the proteins that they were raised against, but *P*. *vivax* culture and invasion assays are not available for routine use. To test an alternative approach, we sought to replace *P*. *knowlesi* target genes with their *P*. *vivax* orthologues, generating chimeric *P*. *knowlesi* strains expressing *P*. *vivax* proteins. Replacement constructs were created in which the *Pv12*, *Pv41*, *PvGAMA* and *PvARP* open reading frames were created by gene synthesis to be recodonised and flanked by the 5’ and 3’ UTRs of their *P*. *knowlesi* counterparts, and these were transfected in combination with the same Cas9/gRNA vectors used in the knockout studies, in order to replace *Pk12*, *Pk41*, *PkGAMA* and *PvARP* with *Pv12*, *Pv41*, *PvGAMA* and *PvARP* respectively (S9A Fig). After selection of transfected parasites with 100 nM pyrimethamine and expansion of the resulting parasites lines, genomic DNA was extracted for genotyping. All lines gave bands of the expected size (S9B Fig) indicating that integration of these replacement constructs had occurred at the expected locus, and no WT bands were detected. Whole genome sequencing analysis confirmed replacement as no reads mapped at the targeted region (S10–S13 Figs) because the Pv gene sequence had been codon-optimised to the human genome to minimise the chance of recombination within the coding sequence, and restrict crossover to the flanking regions, thereby enabling complete gene replacement.

**Fig 6:**
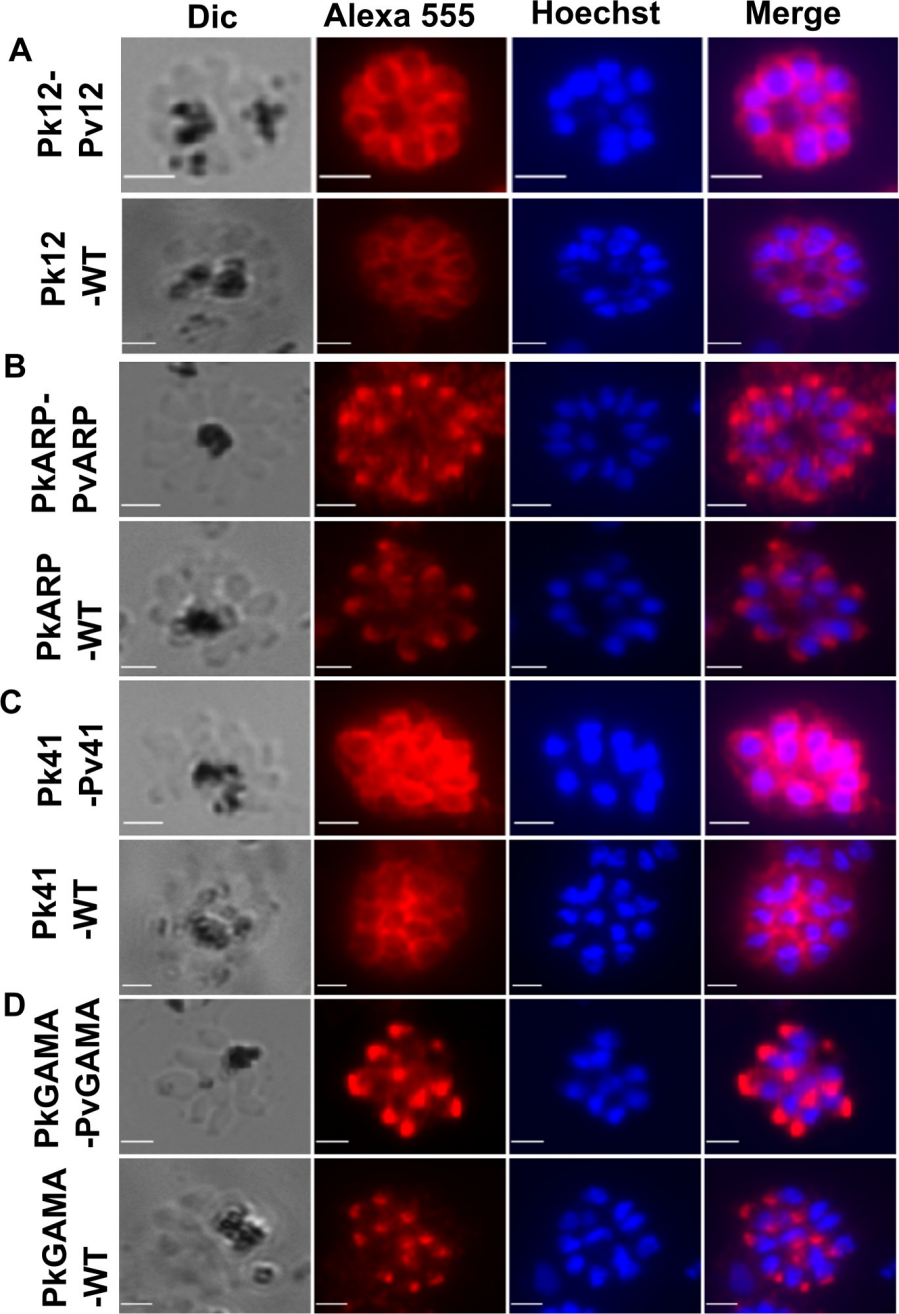
Gene editing to replace *P*. *knowlesi* target genes with orthologous *P*. *vivax* candidate genes. Pvp12, PvARP, Ppv41 and PvGAMA gene sequences were used to replace Pkp12, PkARP, Pkp41 and PkGAMA in wild-type *P*. *knowlesi* to create lines Pk12Rep, PkARPRep, Pk41Rep and PkGAMARep allele replacement lines. Proteins in allele-replacement and wild-type *P*. *knowlesi* strains were localised using anti-*P*. *vivax* polyclonal antibodies and Alexa Fluor 555 labelled secondary antibody, and imaged using fluorescence microscopy. Parasite nuclei were stained with Hoechst 33342; Merge is an overlay of Alexa 555 and Hoeschst. In all cases localisation was the same in the wild-type and allele-replacement lines. Scale bar is 2 micrometers.

Localisation assays with anti-Pv12, anti-PvARP, anti-Pv41 and anti-PvGAMA antibodies all gave specific signals in the replacement lines (Fig 6) with anti-Pv12 and Pv41 (Fig 6A and 6C) indicating merozoite surface localisation, while anti-PvGAMA and PvARP (Fig 6B and 6D) appeared as punctate signals, matching the signals in wildtype *P*. *knowlesi* parasites (Figs 2 and 3). These chimeric parasites are therefore viable and able to correctly express and localize Pv12, PvARP, Pv41 and PvGAMA. The chimeric *P*. *knowlesi* strains had similar growth rates as the WT strains, and in the case of Pv41 and PvGAMA, also grew faster than the relevant Pk knockout strains (S8 Fig), indicating that the *P*. *vivax* genes can substitute for the function of their *P*. *knowlesi* counterparts, and emphasizing the phylogenetic relationship between the two parasites.

**Fig 7:**
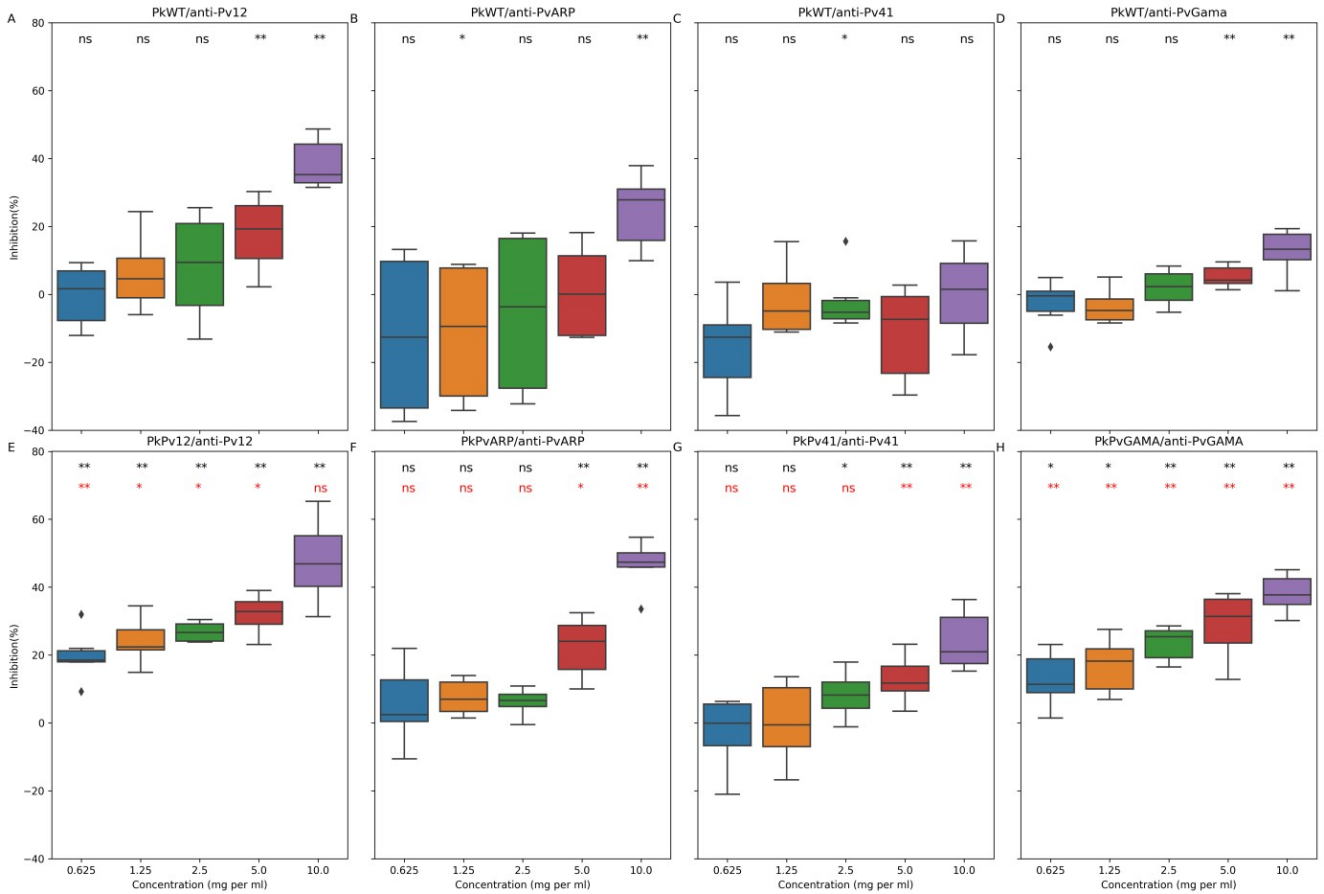
Inhibition by polyclonal antibodies to *P*. *vivax* vaccine candidates is increased in chimeric *P*. *knowlesi* strains expressing homologous *P*. *vivax* proteins. Invasion inhibition assays were carried out as detailed in the Materials and Methods sections and summarised in S4 Fig. Chimeric *P*. *knowlesi* strains expressing Pvp12 (PkPv12), PvARP (PkPvARP), Pvp41 (PkPv41) and PvGAMA (PkPvGAMA) were incubated with anti-PvP12, anti-PvARP, anti-PvP41 and anti-PvGAMA antibodies respectively and compared to inhibition in wildtype *P*. *knowlesi*. This data represents observations from two independent experiments each with three replicate wells per each treatment. To determine if the inhibition was statistically significant, all treatments were independently compared to the same concentration of total IgG from non-immunised rabbits using Mann-Whitney-Wilcoxon test with a p-value threshold of 0.05; the inhibition of wild-type and chimeric parasites was also compared using the same approach. Black asterisks represents p-values obtained from comparison with non-immune rabbit IgG while red asterisks represents p-values obtained from comparison of wild-type and mutant parasites. *p = 0.05–0.01; **p<0.01.

To test whether replacing the *P*. *knowlesi* genes with their *P*. *vivax* counterparts increased the inhibitory activity of anti-*P*. *vivax* antibodies, we tested for invasion inhibition comparing WT and chimeric replacement lines. In all cases inhibition the chimeric allele-replaced lines were inhibited more effectively with the anti-*P*. *vivax* antisera, resulting in lower IC50 values (Figs 7 and S14). This suggests that while *P*. *knowlesi* is a useful model as a first screen for *P*. *vivax* reverse vaccinology studies, sequence differences between *P*. *vivax* antigens and their *P*. *knowlesi* orthologues can lead to underestimation of the inhibitory effect of anti-*P*. *vivax* antibodies when only wildtype *P*. *knowlesi* parasites are used. However, the IC50 values for wildtype *P*. *knowlesi* was lower in these assays than those we had performed previously (Fig 4), raising concern that repeated freeze-thaws had diminished the inhibitory activity of the antibodies. While this technical explanation does not affect comparability between activity on wildtype and allele swap lines within Fig 7 as they were performed at the same time and with the same antibody samples, to address any concerns about reproducibility, we generated new rabbit polyclonal antibodies for Pv12, Pv41, PvGAMA and PvDBP. The IC50 values (3.9, 3.0, 3.2 and 4.4 mg/ml respectively, S15B Fig) from these new antibodies were broadly similar to those generated in Fig 4 (4.5, 5.6, 6.1 and 4.2 mg/ml respectively), confirming that while the absolute values may differ between experiments and different antibody batches, these targets reproducibly induce antibodies with similar GIA IC50s to PvDBP. We also generated Fabs from these new antibodies and confirmed that they too had GIA activity (S15A Fig), which in the case of anti-Pv12 and Pv41 was similar to inhibition by total IgG, reducing concern about possible bivalent effects of IgG.

## Discussion

To date only a limited number of *Plasmodium vivax* blood stage vaccine candidates have been investigated [5,6]. This is in large part because it is currently not possible to continuously culture *P*. *vivax* blood stages *in vitro*, which rules out many biological assays. *P*. *knowlesi* has a close phylogenetic relationship with *P*. *vivax* and has been adapted to *in vitro* culture in human erythrocytes [40,41]. As a result, *P*. *knowlesi* has been recently tested as an *in vitro* model to screen for *P*. *vivax* vaccine candidates, both by screening wild-type human-adapted *P*. *knowlesi* with antibodies raised against recombinant *P*. *vivax* proteins [42,43] and in the case of PvDBP, using CRISPR-Cas9 genome engineering to replace the *PkDBPα* locus with *PvDBP* to facilitate detailed dissection of this major vaccine candidate [46,47]. In this study we combined these approaches, both screening antibodies raised to *P*. *vivax* antigens against wild-type *P*. *knowlesi* parasites, and using systematic genome modification to both delete and swap *P*. *knowlesi* genes for their *P*. *vivax* orthologues, in order screen for new blood-stage *P*. *vivax* antigens using a panel of polyclonal antibodies generated against candidates from a previously published library of *P*. *vivax* schizont expressed proteins [31]. We focused on seven *P*. *vivax* targets: two merozoite surface proteins (PvMSP7.1 and PvMSP3.10); two 6-Cysteine domain proteins (Pv12 and Pv41) and three proteins not belonging to other families (PvGAMA, PvCyRPA and PvARP), with PvDBP included as a positive control.

There was a broad correlation between the ability of anti-*P*. *vivax* antibodies to specifically recognise their *P*. *knowlesi* orthologues in immunoblot and immunofluorescence assays and the percent identity within the *P*. *vivax/P*. *knowlesi* antigen pairs. However, it is worth noting that a single dominant protein band was identified in 5/8 cases, and clear intracellular localisations defined in 7/8 cases, despite levels of identity as low as 50%, suggesting the system has broader utility than primary amino acid homology levels alone might indicate. This high level of cross-reactivity may be in part be due to our strategy of raising polyclonal antibodies against full-length protein ectodomains, whereas many antigen studies focus only on smaller sub-domains, which limits the chances that cross-reactive responses will be generated. There is also some evidence that the eukaryotic expression system we use increases the likelihood of generating antibodies against folded, functional domains [48], which are more likely to be of utility in assays such as immunofluorescence or growth inhibition, where conformation-dependent epitopes are more important. Using these antibodies in growth inhibition assays revealed robust dose-dependent inhibition of *P*. *knowlesi* growth by anti-Pv12, Pv41 and PvGAMA antibodies, in some cases on a similar level to anti-PvDBP antibodies. As with all studies using polyclonal antibodies, some caution about the potential for cross-reactivity with other targets is required, although we do not believe this is a major issue here as in all cases immunofluorescence staining was primarily at expected locations, suggesting either cross-reaction was minimal or occurring only with proteins located in the same organelles as the primary target, which seems unlikely. In the case of two antibodies, anti-Pv41 and anti-PvGAMA, deletion of their respective *P*. *knowlesi* orthologues eliminated all antibody staining, establishing that if cross-reaction was occurring it was below the detection limits of our microscopy assays. Nonetheless, if any of these targets is pursued further, more definitively target-specific reagents such as monoclonal antibodies or affinity-purified antigen-specific antibodies are clearly required.

Pv12 and Pv41 are members of a family of 6-cysteine domain proteins, other members of which are under investigation as transmission-blocking vaccine targets in *P*. *falciparum* [55–57]. The *P*. *falciparum* orthologues of Pv12 and Pv41 form a heterodimer and are localised on the merozoite surface [58]. We have previously shown that Pv12 and Pv41 are also able to heterodimerize [31], and here we show that their *P*. *knowlesi* orthologues also colocalise to the merozoite surface, suggesting key elements of the function of these two proteins are conserved across *Plasmodium* species. However, there are also elements that are different. Immunoepidemiology studies show that antibody responses to Pv12 and Pv41 are commonly induced by exposure to *P*. *vivax* infection and have been associated with protection against severe *P*. *vivax* malaria, in keeping with the inhibitory activity of anti-Pv12 and Pv41 antibodies shown here [31,59–61]. By contrast, in *P*. *falciparum*, anti-Pf12 and Pf41 antibodies have no inhibitory effects on parasite growth *in vitro*, and the genes can be deleted, suggesting a level of functional redundancy in this species [58]. Previous immunofluorescence studies of Pv12 in *P*. *vivax* suggest that unlike its *P*. *falciparum* homologue Pf12 and our experiments with *P*. *knowlesi* it localises to the rhoptries rather than merozoite surface [62]⁠, although whether this apparent difference is due to differences in the stage of the parasite during erythrocytic schizogony used in the assays is not known.

We localised PkARP to the apical region of the merozoite, suggesting a possible location in the rhoptries like the PfARP homologue in *P*. *falciparum* [63], but in contrast to previous studies suggesting a merozoite surface location in *P*. *vivax* and *P*. *knowlesi* [64,65]; again, experimental differences in the stage of parasites used might explain the different observations. PkGAMA also localised to apical organelles, replicating previous observations of a micronemal location in *P*. *vivax* [28] and *P*. *falciparum* [66,67]. We were unable to inhibit *P*. *knowlesi* growth using anti-PvARP antibodies, contradicting what has been shown in *P*. *knowlesi* [65] and *P*. *falciparum* [63]. However, when we replaced PkARP with PvARP, there was a reversal of the activity of anti-PvARP, suggesting that PkARP may lack key inhibitory epitopes recognised by our polyclonals, which were raised against PvARP. Anti-PvGAMA had invasion and growth inhibitory effects on *P*. *knowlesi* in both WT and chimeric lines, replicating the observations of anti-PfGAMA effects on *P*. *falciparum* [66], suggesting a conserved role of this protein in invasion across species. Immunoepidemiology studies also show that antibody responses to PvARP and PvGAMA are commonly induced by exposure to *P*. *vivax* infection and in some cases have been associated with protection against severe *P*. *vivax* malaria [31,59–61,68]⁠.

How do these relatively new candidates compare to the much more well-studied vaccine candidate PvDBP? Clearly PvDBP has been the subject of decades of work, meaning that there are multiple lines of evidence supporting its candidacy. One well-established concern about PvDBP is that some regions of the protein are polymorphic and can induce strain-specific antibody responses [69,70], although extensive work has shown that the issue of polymorphism may be able to be overcome with epitope engineering [14,71,72]. In addition, Phase I trials show that it is possible for a PvDBP immunogen to elicit cross-reactive antibodies that inhibit multiple diverse PvDBP alleles and strains [12,15,47], and cross-reactive human antibodies to PvDBP can be isolated from individuals previously exposed to *P*. *vivax* [73]. PvDBP clearly therefore remains a high priority vaccine candidate, but past experience with *Plasmodium* vaccines suggests that relying only on a single candidate leaves vaccines vulnerable to immune escape, meaning a systematic and comprehensive approach to identify additional candidates is essential, such as the one we pilot here.

One question in weighing up the candidacy of any new vaccine antigen is whether the gene that encodes it is essential for parasite growth, as targeting a non-essential gene would seem likely to select for parasites that do not rely on the gene product, and so are able to escape the vaccine. According to the current model, PvDBP is essential for invasion, as *P*. *vivax* primarily invades via the interaction between PvDBP and its host receptor, Duffy Antigen Receptor for Chemokine (DARC) [17–20]. While it has now clearly been demonstrated that *P*. *vivax* is also able to infect individuals who are Duffy negative, lacking DARC expression on their red blood cell surface [21–23], this could either be because *P*. *vivax* is able to utilize other ligands for invasion such as PvRBP2b [27] and erythrocyte binding protein 2 (ebp2) [29], or that PvDBP is still involved in the invasion of Duffy negative cells [24,25]. It is also worth noting that the genetic essentiality of *PvDBP* for parasite growth has never been able to be directly tested, as *P*. *vivax* cannot be cultured and therefore cannot be genetically manipulated. Studies in *P*. *knowlesi*, which has three homologues of *PvDBP*, suggest that at least one is required for invasion of human erythrocytes [46,54], but whether this is true of *PvDBP* remains to be proven unequivocally.

In the case of the four candidates identified by initial screening with wildtype *P*. *knowlesi*, there are clear 1:1 orthologues between *P*. *vivax* and *P*. *knowlesi*, providing an strong rationale to use *P*. *knowlesi* genetic tools to explore candidates. We utilised the fact that *P*. *knowlesi* can be readily genetically manipulated [40,44–46] to explore whether Pv12, Pv41, PvGAMA and PvARP were essential for parasite growth. *Pk41* and *PkGAMA* could be experimentally deleted, while *Pk12* and *PkARP* could not, even though both anti-PvP41 and PvP12 antibodies had invasion and growth inhibitory effects on WT *P*. *knowlesi*. The fact that *Pk12 and PkARP* could not be deleted using this strategy does not of course confirm that these genes are essential for blood stage growth, as different constructs or a different strategy could have had a different outcome. However, three different gRNA constructs were used for these as for every gene, which does decrease the likelihood that ineffective Cas9 cleavage alone is an explanation for the fact that *Pk12* and *PkARP* could not be readily deleted.

The fact that *Pk41* and *PkGAMA* could be deleted, whereas antibodies that recognise them inhibit growth, at first glance seems contradictory, even though there was a small but statistically significant growth defect in both knockout lines suggsteing some role for these genes during routine blood-stage growth. The difference between the antibody and knockout data could be explained if the antibodies raised against Pv41 and PvGAMA recognized multiple targets in *P*. *knowlesi*, but these antibodies generated only non-specific immunofluorescence staining with the knockout strains, suggesting that the antibodies are specific. An alternative explanation is that the process of genetic deletion, which takes some weeks to recover modified parasites, provides an opportunity for the parasites to adapt to the loss of a specific gene, for example by up-regulating the expression of other genes. By contrast, growth inhibition assays using antibodies occur in a single cycle, which the parasites may find it harder to adapt to. There is certainly precedence for differences between antibody inhibition and genetic deletion data from the study of invasion-associated proteins in *P*. *falciparum*. Antibodies raised against PfEBA175, for example, are able to inhibit invasion in multiple *P*. *falciparum* strains [74,75], yet the *PfEBA175* gene can be deleted without affecting parasite growth [76] and in some cases gene deletion has been shown to be accompanied by a transcriptional switch in gene expression which presumably helps the parasites to survive in the absence of PfEBA175, indicative of the kind of adaptation approach that can happen during genetic manipulation experiments [77]. Similarly anti-Pf12 and anti-Pf41 antibodies were able to inhibit *P*. *falciparum* growth, albeit at low levels (15–20%), yet both genes could be disrupted [58]. Genetic deletions and inhibitory antibodies are different model approaches to understand protein function, each with strengths and weaknesses. Overall though, the lack of genetic essentiality for Pv41 and PvGAMA does make it reasonable to argue that Pv12 and PvARP should have a higher priority for follow-up.

This study clearly highlights several advantages of the *P*. *knowlesi* system as a model for testing *P*. *vivax* blood-stage antigens, some of which have also been emphasised in previous drug [78] and vaccine [42,43,46,47,65] studies. A key one is accessibility—access to *ex vivo P*. *vivax* samples is limited and samples are precious, whereas we were readily able to perform multiple *in vitro* assays using a human-erythrocyte adapted line of *P*. *knowlesi* [40]. A second is the genetic tractability of the system, where transfections, gene deletions and allele replacements, while not precisely routine, are certainly readily achievable [44–46,78]. There are however always limitations in using one species as a model for another, and this study reveals some of these. The study relies on antibodies that were generated against *P*. *vivax* proteins being able to cross-react with their *P*. *knowlesi* orthologues, and while in almost all cases this proved possible, there was some correlation between the strength of cross-reactivity and the % identity between antigen pairs, meaning the *P*. *knowlesi* model will almost certainly be more useful for some antigens than others. The genetic tractability of *P*. *knowlesi* offers a potential solution to this problem, as we have shown, by allowing the replacement of endogenous *P*. *knowlesi* genes with their *P*. *vivax* orthologues, implying that antibodies can be tested against the precise sequence they were generated against. This approach relies on the ability of a *P*. *vivax* gene to substitute for *P*. *knowlesi* gene function, which may not always be the case, but in all four instances tested here, as well as the Pv/PkDBP swaps carried out previously (where the Pv/PkDBP swap behaved very similarly to *ex vivo P*. *vivax* assays, and much better than assays using recombinant proteins) [46,47], this has not proven a problem, despite in the case of PvDBP/PkDBPα, relatively low levels of overall homology. Ultimately however, no model system is perfect, even *ex vivo* culture of *P*. *vivax* itself, which after all is only a model for *in vivo* growth. It would be extremely useful to the *P*. *vivax* vaccine field to carry out a head-to-head comparison across all the four currently available functional models—wildtype *P*. *knowlesi*, genetically modified *P*. *knowlesi* with allele modifications to insert *P*. *vivax* genes, *P*. *cynomologi* and *P*. *vivax ex vivo* assays. The targets identified here, Pv12, Pv41, PvARP and PvGAMA, along with PvDBP, present a perfect opportunity to carry out such a test. It is also worth noting that genetically modified lines such as those generated here could also prove very useful for immunoepidemiological work, such as using them to test the inhibitory activity of antibodies purified from *P*. *vivax* exposed patient sera.

To conclude, using both antibody and genetic approaches, we exploited the phylogenetic relationship between *P*. *knowlesi* and *P*. *vivax* to explore blood-stage *P*. *vivax* vaccine targets. The data suggests a hierarchy of possible targets, with Pv12 and PvARP being the highest priority as they appear to be genetically essential and can be targeted with inhibitory antibodies, Pv41 and PvGAMA in a second tier as they can be inhibited with antibodies but also genetically deleted, while PvMSP7.1, PvMSP3.10 and PvCyRPA would seem to have the lowest priority, at least on the basis of the assays we performed. There are of course caveats to this kind of ranking, as with any ranking. Firstly, we did not pursue PvMSP7.1, PvMSP3.10 and PvCyRPA in detail after antibodies against them did not yield inhibitory activity in initial experiments, which could potentially be due to low levels of homology between *P*. *vivax* and *P*. *knowlesi* in these proteins. In the case of PvCyRPA, encouraging data from *P*. *falciparum* [79] and the finding that *PkCyRPA* is essential for *P*. *knowlesi* invasion [45] both suggest that further investigation of this target is justified. Secondly, comparing antibody inhibition on the basis of total IgG IC50 has its limits—generating antigen-specific antibodies for each target would be a much more accurate basis on which to carry out comparisons, but was not possible in this study due to the sheer number of different assays and approaches being employed. Detailed follow-up should inevitably involve the generation of monoclonal antibodies for more datailed mechanism of action studies.

The overall approach reinforces other studies [42,43,46] to suggest that *P*. *knowlesi* can serve as an accessible and efficient model to test *P*. *vivax* vaccine candidates. While this offers a promising route to scale-up *P*. *vivax* blood-stage vaccine candidate screening, as noted above there are advantages and limitations to any model system. Until a robust *P*. *vivax* culture system is established, which despite extensive effort by multiple groups [33–37]⁠ does not seem likely soon, it will be advisable to use multiple models to screen for candidates, and be clear and upfront about the limitations inherent in all of them.

## Materials and methods

### Ethics statement

Human O+ erythrocytes were purchased from NHS Blood and Transplant, Cambridge, UK, and all samples were anonymised. The work complied with all relevant ethical regulations for work with human participants. The use of erythrocytes from human donors for *P*. *falciparum* culture and binding studies was approved by the NHS Cambridgeshire 4 Research Ethics Committee (REC reference 15/EE/0253) and the Wellcome Sanger Institute Human Materials and Data Management Committee.

### *In vitro* parasite culture

*Plasmodium knowlesi* parasites were maintained as described in [40]. Briefly, *P*. *knowlesi* strain A1-H.1 was propagated in human O^+^ erythrocytes (UK NHS Blood and Transplant), in RPMI 1640 supplemented with Albumax (Thermo Fisher Scientific), L-Glutamine (Thermo Fisher Scientific), Horse serum (Thermo Fisher Scientific), Gentamicin (Thermo Fisher Scientific). The cultures were kept at 2% hematocrit, gassed using a mixture of 5% CO_2_, 5% O_2_ and 90% Nitrogen, while being monitored three times per week by counting parasitemia using light microscopy with media change or splitting as appropriate.

### Synchronization and enrichment of *Plasmodium knowlesi*

Synchronization was performed by enriching late stage parasites using Histodenz (Sigma Aldrich) as described in [40]. Briefly, parasites were resuspended in 5ml complete media and layered on top of 5 ml of 55% Histodenz in complete culture media in a 15 ml tube (Greiner). The mixture was then centrifuged for 3 minutes at room temperature, 1500 g, acceleration 3 and brake 1, resulting in late stage parasites becoming enriched at the interface. For inhibition assays, these parasites were returned to culture, and assays set up after reinvasion had occurred in the subsequent cycle. For protein extracts, immunofluorescence assays and transfections, this was repeated over three consecutive cell cycles to create very tightly synchronized parasites, with schizont samples from a fourth cycle of Histodenz purification used for subsequent analysis.

### Antibody production and purification

Rabbits were immunized with 1mg of his-tagged *P*. *vivax* full-length ectodomain, expressed in HEK239E cells as previously described [31] (except in the case of repeat immunisations for Pv12, Pv41 and PvARP, where a Cd4 tag included in the initial constructs was not included), and purified by nickel affinity chromatography. Immunisations were carried out using Freunds incomplete (day 0)/Freunds complete adjuvant (days 14, 28, 42, 56 and 70) by Cambridge Research Biochemicals, with a total of 1mg of protein immunised over the course of the immunisation schedule. The total amount of antigen immunised varied between proteins (PvGAMA: 0.52mg, PvP12: 0.87mg, PvMSP3.10: 0.69mg, PvMSP7.1: 0.71mg, PvARP: 3.47mg, PvCyRPA: 1.35mg, PvDBP: 1.35mg, PvP41: 3.57mg), with the total being equally distributed across all immunisations. Total IgG was purified using Protein G gravitrap kit (GE healthcare). Eluted total IgG was concentrated by centrifugation at 4°C for 30 mins using 100000MWCO Vivaspin (Sartorius). The concentrate was then dialyzed using a dialysis tube (Millipore) overnight at 4°C with 1 litre of RPMI 1640 (Thermo Fisher Scientific), before repeating concentration if necessary. Total IgG concentration was measured by nanodrop (NanoDrop). For Fab purification, purified IgG was buffer exchanged in digestion buffer two times in a 5ml 10kDa concentrator (Amicon Ultra-4, Cat. No. UFC801024), followed by running in a desalting column. Fab purification was performed according to the manufacturers protocol (Pierce Fab preparation kit, Cat no.44985), with samples digested for 2 hours at 37 degree with end-to-end rotation at 20 rpm.

### Protein extraction and western blotting

To generate protein extracts, schizont stage parasites enriched from 5–10 mL of culture at 5–10% parasitemia were treated with 0.15% saponin (Sigma Aldrich) and protease inhibitor (Cat. No. 5892970001, Sigma Aldrich) at 1X for 1min on ice to release parasites from their host cell. After pelleting and two rounds of washing in ice cold 1X PBS (Sigma Aldrich) with protease inhibitor 1X, parasites were treated with 1 μl of DNAse I (Thermoscientific) for 30mins at 37°C., before mixing 1:1 with Laemmli sample buffer 2X and incubating for 30mins-1h at 37°C to gently denaturate the sample. The pellet was then frozen down at -80°C until needed. Samples were diluted 0, 1:5 or 1:10 in Laemmli, and 5μl of the diluted samples was mixed with 2.5 μl NuPage LDS sample buffer (4X) (Thermo Fisher Scientific), 1 μl NuPage Reducing agent (10X) (Thermo Fisher Scientific) and deionized water to 6.5 μl. The mixture was then incubated at 72°C for 10 mins while shaking at 300 RPM. 10 μl of the sample was then resolved on a 4–12% bis-trisNuPage gel (Thermo Fisher Scientific) with 1X MOPS SDS gel buffer (Thermo Fisher Scientific) at 200 V for 50 minutes.

Western blot transfer was carried out in wet conditions at 30V for 60min, and membrane blocked overnight while shaking at 4°C in 5% milk (Marvel) containing 0.077% sodium azide (Sigma Aldrich). Primary antibodies were diluted in 5% milk/PBS/0.1% TWEEN 20 at concentrations as follows: anti-PvGAMA 1:2400; Pvp12, PvMSP7.1 and PvMSP3.10 1:400; Pvp41, PvARP and PvCyRPA 1:800; PvDBP 1:1600. Primary incubation was carried out overnight at 4°C. Blots were then washed three times, each 10mins, in PBST (1X PBS and 0.1% Tween), before incubating with secondary anti-Rabbit HRP (Abcam) at 1:20000 dilution in 5% milk/PBS/0.1% for 45 mins at room temperature. Blots were washed again three times, each 10mins, in PBST before developing with ECL prime Western Blotting detection reagent, (GE Healthcare). Expected molecular weight of the *P*. *vivax* candidate proteins and their orthologous proteins in *P*. *knowlesi* was predicted using Protein Molecular Weight calculator (available at https://www.bioinformatics.org/sms/prot_mw.html) based on the amino acid sequences of the respective protein sequences from PlasmoDB.

### Immunofluorescence assays

Cells were synchronized, harvested from culture and enriched using Histodenz as described above based on [40]. These were then washed with 1X PBS (Sigma Aldrich) for 5 mins and fixed with 4% paraformaldehyde (Agar scientific)/ 0.0075% glutaraldehyde (Sigma Aldrich) in 1X PBS for 30 minutes at room temperature, followed up with washing in 1x PBS while shaking for 5 mins. Thin smears of fixed cells were made on Poly-L-Slides (Sigma Aldrich) and stored in -80°C freezer until needed. For processing, slides were incubated briefly at room temperature, permeabilized with 0.1% Triton X-100 (Sigma Aldrich) in 1X PBS for 30 mins at room temperature then washed once with 1x PBS while shaking for 5 mins. Blocking was carried out overnight at 4°C in a humidified dark chamber using Blocking Aid Solution (Thermo Fisher Scientific). Co-localising antibodies were obtained for *P*. *falciparum* PfGAP45 [80] which we have shown cross-reacts with *P*. *knowlesi*, PfAMA1 (obtained through BEI Resources, NIAID, NIH: Monoclonal Anti-*Plasmodium* Apical Membrane Antigen 1, Clone 28G2 (producted *in vitro*), MRA-897A, contributed by Alan W. Thomas) which is reported to cross-react with all *Plasmodium* species, and for *P*. *knowlesi* PkMSP1 [81]. Primary antibody diluted in Blocking Aid Solution (Anti-PvGAMA 1:1200; Pvp12, PvMSP7.1 and PvMSP3.10 1:200; PvARP 1:400; PvCyRPA and PvDBP 1:800; Pvp41 1:200, PkMSP1-19 1:2000; PfAMA1 1:1000) was then added and incubated overnight in a humidified dark chamber at 4°C followed by washing three times for 5 mins in 1X PBS while shaking. Secondary antibody diluted in Blocking Aid Solution (Alexa Fluor 555 Goat-anti rabbit (Thermo Fisher Scientific) (1:500) for anti-*P*. *vivax* rabbit polyclonals and Alexa Fluor 488 Goat-anti rat (Thermo Fisher Scientific) (1:500) for PkMSP1-19 and PfAMA1) was then added and incubated 1 hour in a humidified dark chamber at room temperature followed by washing three times for 5 mins in 1X PBS while shaking. Hoechst 33342 (Thermo Fisher Scientific), for nucleus staining, was diluted 1:3000 in 1x PBS (Sigma Aldrich) then added and incubated for 10 mins in a humidified dark chamber at room temperature with subsequently washing three times for 5 mins in 1X PBS while shaking. The cells were later mounted with Pro-Long Gold mounting solution (Thermo Fisher Scientific), covered with cover-glass (VWR), left to cure for 24 hours in dark and dry chamber at room temperature and eventually sealed with slide sealer (Biotium), before imaging on a Leica DMi8.

### Invasion assays

Two millilitres of O+ erythrocytes at 2% hematocrit in incomplete culture media were labelled using 2 μl of a stock of 1 mM Far-red Cell Trace (Thermo Fisher Scientific); control unstained erythrocytes were incubated with 2 μl of Dimethyl-sulphoxide (DMSO; Sigma Aldrich). After 2 hours of incubation at 37°C while shaking, labelled erythrocytes were washed twice using complete media, then the cells were resuspended in complete media to 2% hematocrit in 2 ml final volume. For test samples, labelled erythrocytes were mixed with synchronized rings at 4% parasitemia (generated by Histodenz synchronisation as described above) and serial dilutions of anti-*P*. *vivax* antibodies(with all dilutions made using incomplete medium), while for controls samples, the same components were mixed except no antibodies were used rather only incomplete media was added Each test sample therefore contained 20 μl infected erythrocytes, 20 μl stained erythrocytes, Xμl of diluted total IgG (Xμl because the antibodies were in varying stock concentrations therefore requiring different volumes to be added to get the same final concentrating). Control wells contained 40 μl of erythrocytes only, or 20 μl of infected erythrocytes/20 μl of unstained erythrocytes, or 40 μl of stained erythrocytes only, or 20 μl of infected erythrocytes and 20 μl of stained erythrocytes, to control for gating in the flow cytometry. Finally all the above treatments were supplemented with 2.2 μl of a mixture of serum, hypoxanthine and gentamicin (at a ratio of 2, 0.18 and 0.009 respectively). Triplicates of each combination were incubated in a 96 well plate for 24 hours in a gassed chamber. To quantify parasite invasion, samples were centrifuged for 3 mins at 450g (acceleration 9, brake 3) at room temperature, supernatant was removed and samples were labelled with SYBR green I nucleic acid dye (Thermo Fisher Scientific) for 1 hour at 37°C while shaking at 52 rpm. After two washes with 100 μl 1 X PBS (Sigma Aldrich), samples were resuspended in 100 μl of 1 X PBS (Sigma Aldrich) and parasites quantified using FACS (Cytoflex, Becton and coulter) as previously described [82]. Data was analyzed using FlowJo then using Excel, invasion was calculated as the percentage of erythrocytes that were both SYBR green and Far-red Cell Trace positive as compared to only DMSO treated erythrocytes. The results were then plotted using the following Python packages: Seaborn [83], Matplotlib [84], Pandas [85], statannot [86] in PyCharm Community Edition (JetBrains s.r.o.). IC50 was determined using Robust linear regression model (RLM) using the Python package statsmodels [87].

### Genetic modification

#### Gene repair construct design and assembly

Constructs, guide RNAs and primers (S1–S6 Tables) were designed with [88] and [89]. Synthetic DNA codon optimization was performed using gblocks Gene Fragments (IDT) (PvGAMA_regions1 & 3, PvARP_region1) and GeneArt Gene Synthesis (Thermo Fischer Scientific) (Pv12, Pv41, PvARP_region3). PvGAMA_regions2 and PvARP_regions3 were amplified from expression constructs previously generated by [31]⁠. Other fragments were amplified from *P*. *knowlesi* genomic DNA, purified from saponin-lysed *P*. *knowlesi* infected erythrocytes using DNA blood kit (Qiagen) according to the manufacturer’s protocol. gRNA and gene knockout vectors were assembled in vectors kindly provided by the Moon lab [46], and allele replacement vectors were assembled in PUC19 using Gibson assembly according to the manufacturer’s protocol (NEB), using PCR products amplified using KAPA HiFi HotStart ReadyMixPCR Kit (KAPABiosystems) and purified using gel isolation kits (Macherey and Nagel). Primers (S1 and S2 Tables) and PCR programs (S3 Table: KAPA2M for all of constructs except KAP121M for final amplification of *Pkgama* replacement insert) are listed in the Supplementary Material.

#### Assembly of Cas9/gRNA vectors

The cloning vector (pCas9/sg_GOI [46]) was digested using BtgZI (NEB), purified using a gel purification kit (Macherey and Nagel), and treated with shrimp alkaline phosphatase (NEB) to dephosphorylate vector ends. Guide RNAs (S5 Table) were reconstituted by mixing 1 μl of 100 μM stocks of the forward and reverse strands for each guide with 1 μl of 10x ligation buffer (NEB), 0.5 μl T4 polynucleotide kinase (NEB) and 65 μl nuclease free PCR water. Annealing was carried out by incubating at 37°C for 30 min, then increasing to 94°C for 5 min before cooling at 25°C at a ramp speed of 5°C per-min. Annealed primers were then diluted to 1 μl in 200 μl and ligated (NEB) to the digested and dephosphorylated Cas9 vector. Three gRNAs were constructed for each gene target.

Vectors were transformed into chemically competent *E*. *coli* according to the manufacturer’s protocol (NEB), and grown overnight. Resulting colonies were screened by colony PCR using GoTaq Green PCR master mix (Promega), with 1 μl of Pk5’ UTR forward and Pk3’UTR reverse primers for each respective construct. Positive colonies were expanded and DNA purified using a miniprep purification kit (Macherey and Nagel) and sequenced to confirm construct integrity (GATC/Eurofins). Sequencing data was analyzed using Benchling and Seqman Pro (DNA star Navigator); positive constructs were expanded and purified for transfection using a maxiprep purification kit (Macherey and Nagel).

### Transfection

Transfection was performed largely as described in [40]. Late stage *P*. *knowlesi* parasites were enriched using Histodenz as described above. In each transfection cuvette (Lonza), 10 μl of schizonts was mixed with 100 μl of P3 solution (Lonza) containing 30 μg each of the relevant donor and three guide vectors. Transfections were carried out using program FP158 (Amaxa Nucleofector, Lonza), and the contents were then immediately transferred into a 2 ml sterile eppendorf tube containing 500 μl of complete culture media mixed with 190 μl uninfected erythrocytes. The transfection mix was incubated at 37°C while shaking at 800 rpm in a thermomixer for 30mins, before being transferred into a 6 well plate, gassed and incubated at 37°C for one parasite life cycle. After this selection was applied with 100 nM pyrimethamine (Santa Cruz Biotechnology Inc). For three days, the cultures were monitored by smearing and selection done by changing the media and replacing with fresh media containing 100 nM pyrimethamine (Santa Cruz Biotechnology Inc). On day 4 post transfection the cultures were diluted 1/3 in 5ml fresh media containing 100 nM pyrimethamine and 100 μl erythrocytes. The cultures were then maintained and monitored after every 2nd cycle by smearing/parasitemia counting, with media changed or cultures split as appropriate. Samples where parasites re-appeared were expanded in a total volume of 50 ml with erythrocytes at 2% hematocrit until parasitemia was greater than 5%. DNA was then isolated using a DNA Blood kit (Qiagen) and analysed using gene-specific primers (S6 Table) and PCR program KAPA18C (S3 Table). Cultures that contained only modified parasites were phenotyped without cloning, while those that genotyping showed had both modified and WT genotyping bands were cloned by limiting dilution and plaque cloning in flat-bottomed 96-well plates. Wells containing single plaques were identified using an EVOS microscope (4x objective, transmitted light), expanded, and DNA isolated and genotyped as described above as well as whole genome sequenced. Whole Genome Sequencing analysis was performed on the Welcome Sanger Institute Cluster using bowtie2 [90] and samtools [91]. Visualisation was performed using Integrative Genomics Viewer [92] as described in [93].

### Growth rate assays for genetically modified lines

Genetically modified *P*. *knowlsei* strains as well as the wild-type control were synchronised using Histodenz. On Day 0 these cultures were diluted to 4% parasitemia of tightly synchronised rings then mixed with equal volumes of uninfected erythrocytes. These were then supplemented with 2.2 μl of a mixture of serum, hypoxanthine and gentamicin (at a ratio of 2, 0.18 and 0.009 respectively). Triplicates of each combination were incubated in a 96 well plate for 24 hours in a gassed chamber. After these 24 hours i.e. Day 1, the number of infected cells was determined as following; samples were centrifuged for 3 mins at 450g (acceleration 9, brake 3) at room temperature, supernatant was removed and samples were labelled with SYBR green I nucleic acid dye (Thermo Fisher Scientific) for 1 hour at 37°C while shaking at 52 rpm. After two washes with 100 μl 1 X PBS (Sigma Aldrich), samples were resuspended in 100 μl of 1 X PBS (Sigma Aldrich) and parasites quantified using FACS (Cytoflex, Becton and coulter) as previously described [82]. Parasitemia was then quantified as the number of cells that were SYBR green positive as a percentage of the number of cells measured. After quantification, cultures were split and diluted to equal parasitemia. This process was repeated over a total of 19 cycles. Data was analyzed using FlowJo (FlowJo) and Excel (Microsoft Office). Growth rate was quantified by dividing the parasitemia obtained before splitting in the current cycle with parasitemia obtained after splitting on the previous cycle, and average growth rate calculated over the entire 19-cycle experiment. The results were then plotted using the following R packages; ggplot2 [94], ggpubr [95], cowplot [96], magrittr [97], readxl [98] and dplyr [99] in R-Studio (R-Studio Inc).

## Supporting information

S1 Fig Preimmune antibody controls for immunolocalization. Localisation of using pre-immune serum from rabbits before they were immunized with PvGAMA, PvDBP, PvARP, PvCyRPA, PvMSP7.1 and Pv41, respectively. No specific staining is detected using Alexa 555-labeled anti-rabbit secondary antibody. Merge is an overlay of Alexa 555 and Hoeschst (parasite nuclei). Scale bar is 2 micrometers.
(TIFF)

S2 Fig Colocalization of *P*. *knowlesi* proteins using polyclonal anti-*P*. *vivax* antibodies to *P*. *vivax* vaccine candidates with antibodies to PkMSP1 and PfGAP45. *P*. *knowlesi* proteins were localised in schizont and trophozoite (troph) stage parasites using rabbit polyclonal antibodies raised against Pv12, PvMSP7.1 and Pv41 and co-stained with rat antibodies against A) anti-PkMSP1 or B) anti-PfGAP45 for colocalization (origin of antibodies is described in Methods). Alexa Fluor 555 goat-anti rabbit and Alexa Fluor 488 goat-anti rat were used as secondary antibodies, and parasite DNA localised using Hoechst 33342. The first Merge column is one is an overlay of Alexa 555 and Alexa488 staining, while the second Merge column is an overlay of Alexa 555, Alexa488 and Hoeschst. Scale bar is 2 micrometers.
(TIFF)

S3 Fig Colocalization of *P*. *knowlesi* proteins using polyclonal anti-*P*. *vivax* antibodies to *P*. *vivax* vaccine candidates with antibodies to proteins PkMSP1 and PfAMA1. *P*. *knowlesi* proteins were localised in schizont stage parasites using rabbit polyclonal antibodies raised against *P*. *vivax* vaccine candidates and co-stained with rat antibodies against A) anti-PkMSP1 or B) anti-PfAMA1 for colocalization (origin of antibodies is described in Methods). Alexa Fluor 555 goat-anti rabbit and Alexa Fluor 488 goat-anti rat (MSP1) or mouse (AMA1) were used as secondary antibodies, and parasite DNA localised using Hoechst 33342. The first Merge column is one is an overlay of Alexa 555 and Alexa488 staining, while the second Merge column is an overlay of Alexa 555, Alexa488 and Hoeschst. Scale bar is 2 micrometers.
(TIFF)

S4 Fig Diagram outlining invasion inhibition assays. A) Synchronized *P*. *knowlesi* cultures at ring stage (IRBC) were mixed with Far-Red Cell Trace dye stained Uninfected RBCs (SUIRBC) and two-fold serial dilutions from 10 mg/ml to 0.625 mg/ml of purified total IgG. B) The mixture was incubated for 24 hours under normal culture conditions described in the Material and Methods section to allow a full cycle of development and invasion to occur. C) Parasite DNA was labelled with SYBR Green, and newly invaded parasites identified using two-colour flow cytometry, where new invasions into Far-Red Cell Trace labelled RBCs can be detected in the upper right quadrant (3—circled).
(TIFF)

S5 Fig Gene editing strategy to knock out candidate genes. A) General strategy used to attempt to knock out *Pk12*, *Pkarp*, *Pkdbpalpha*, *Pk41* and *Pkgama*. Plasmids used are Cas9/gRNA vector and donor vector containing eGFP (GFP) flanked with 5’ and 3’ untranslated region (UTR) for each respective *P*. *knowlesi* gene (PkCDS). Primer pairs used for genotyping are P1&P2 and P3&P4, to test for the presence of wildtype gene, and P1&P5 and P6&P4 to test for integration of the knockout construct. B) Genotyping of Pk41KO and PkGAMAKO with above primer pairs as compared to WT. On the right side are the obtained molecular weight in kilobase pairs (kb).
(TIFF)

S6 Fig Whole genome sequencing of Pk41 knockout strain. Reads generated from Illumina sequencing of Pk41 knockout strains and the WT strain from which they were generated were aligned to the *P*. *knowlesi* reference genome. Absence of reads in the centre of the gene confirms deletion of this locus from the *P*. *knowlesi* genome.
(TIFF)

S7 Fig Whole genome sequencing of PkGAMA knockout strain. Reads generated from Illumina sequencing of PkGAMA knockout strains and the WT strain from which they were generated were aligned to the *P*. *knowlesi* reference genome. Absence of reads in the centre of the gene confirms deletion of this locus from the *P*. *knowlesi* genome.
(TIFF)

S8 Fig Comparative Growth rate assay between wildtype *P*. *knowlesi* and genetically edited *P*. *knowlesi strains*. WT (P. knowlesi WT, PkMC), Gamako (PkGama knock-out clone), Gamarep (PkGama replacement clone), p41ko (Pkp41 knock out clone), p41rep (Pkp41 replacement clone), P12rep (Pkp12 replacement clone), and ARPrep (PkARP replacement clone) strains were tightly synchronised, and parasitemia measured every day using flow cytometry, then readjusted to the same parasitemia again. Parasitmia was quantified in this manner for 19 days (ie. over 19 growth cycles) and the average growth rate per cycle calculated for each strain. All strains were grown in triplicate.
(TIFF)

S9 Fig Gene editing strategy to replace *P*. *knowlesi* target genes with orthologous *P*. *vivax* candidate genes. A) General strategy used to replace *pk12*, *pkarp*, *pk41* and *pkgama* with *pv12*, *pvarp*, *pv41* and *pvgama*, respectively. Plasmids used are Cas9/gRNA vector and donor vector containing the *P*. *vivax* coding sequence (PvCDS) flanked with 5’ and 3’ untranslated region (UTR) for each respective *P*. *knowlesi* gene (PkCDS). Primer pairs used for genotyping are P1&P2 and P3&P4, to test for the presence of the wildtype gene. P1&P5 and P6&P4 to test for integration of the replacement construct. B) Genotyping of pk12, pkarp, pk41 and pkgama allele replacement (Rep) using the above primer pairs as compared to WT. On the right side are the obtained molecular weight in kilobase pairs (kb).
(TIFF)

S10 Fig Whole genome sequencing of Pk41-Pv41 replacement strain. Reads generated from Illumina sequencing of PkPv41 allele replacement strains and the WT strain from which they were generated were aligned to the *P*. *knowlesi* reference genome. The *P*. *vivax* gene construct was codon optimised to human codons to reduce the chance that recombination would occur within the ORF, rather than within the flanking regions, and hence favour complete replacement of the *P*. *knowlesi* gene. As a result, reads from the replacement gene do not map to the *P*. *knowlesi* genome, confirming that the endogenous gene has been replaced.
(TIFF)

S11 Fig Whole genome sequencing of PkGAMA-PvGAMA replacement strain. Reads generated from Illumina sequencing of PkPvGAMA allele replacement strains and the WT strain from which they were generated were aligned to the *P*. *knowlesi* reference genome. The *P*. *vivax* gene construct was codon optimised to human codons to reduce the chance that recombination would occur within the ORF, rather than within the flanking regions, and hence favour complete replacement of the *P*. *knowlesi* gene. As a result, reads from the replacement gene do not map to the *P*. *knowlesi* genome, confirming that the endogenous gene has been replaced.
(TIFF)

S12 Fig Whole genome sequencing of PkARP-PvARP replacement strain. Reads generated from Illumina sequencing of PkPvARP allele replacement strains and the WT strain from which they were generated were aligned to the *P*. *knowlesi* reference genome. The *P*. *vivax* gene construct was codon optimised to human codons to reduce the chance that recombination would occur within the ORF, rather than within the flanking regions, and hence favour complete replacement of the *P*. *knowlesi* gene. As a result, reads from the replacement gene do not map to the *P*. *knowlesi* genome, confirming that the endogenous gene has been replaced.
(TIFF)

S13 Fig Whole genome sequencing of Pk12-Pv12 replacement strain. Reads generated from Illumina sequencing of PkPv12 allele replacement strains and the WT strain from which they were generated were aligned to the *P*. *knowlesi* reference genome. The *P*. *vivax* gene construct was codon optimised to human codons to reduce the chance that recombination would occur within the ORF, rather than within the flanking regions, and hence favour complete replacement of the *P*. *knowlesi* gene. As a result, reads from the replacement gene do not map to the *P*. *knowlesi* genome, confirming that the endogenous gene has been replaced.
(TIFF)

S14 Fig Comparison of IC50 resulting from invasion inhibitory assays of *P*. *knowlesi* wild-type and allele replacement mutants using antibodies against *P*. *vivax* proteins. Using the invasion inhibition assay data from Fig 8 above, IC50 in mg/ml was generated using Robust linear regression model. To determine if there were statically significant differences between these values, IC50 values obtained from the allele replacement mutants (Replacements) were independently compared to *P*. *knowlesi* wild-type IC50 (PKWT) that had been treated with the same antibodies using Mann-Whitney-Wilcoxon test with a p value threshold of 5.00e-02. *: 1.00e-02 < p value < = 5.00e-02. **: 1.00e-03 < p value < = 1.00e-02.
(TIFF)

S15 Fig Growth inhibition by total IgG and Fab fragments from a second batch of rabbit polyclonal antibodies. Rabbit polyclonal antibodies were raised against PvDBP, Pv12, Pv41 and PvGAMA as described in the Methods, and used in Growth Inhibition Assays as outlined in S4 Fig using either a single dose of purified Fab (A) or a dose dilution series of total IgG (B).
(TIFF)

S1 Table Primers used for amplification of sequences used for gene knockout construct generation. Contains a list of all primers used for generation of *Plasmodium knowlesi* gene knockout constructs.
(XLSX)

S2 Table Primers use for amplification of sequences used for gene replacement construct generation, Contains a list of all primers used for generation of *Plasmodium knowlesi* gene replacement constructs. (XLSX)

S3 Table List of PCR programmes used for construct generation. Details of PCR cycle temperatures and times for generation of *Plasmodium knowlesi* knockout and gene replacement constructs.
(XLSX)

S4 Table Genetic modification construct sequences. Vector sequences for *Plasmodium knowlesi* knockout and gene replacement constructs.
(XLSX)

S5 Table Guide RNA sequences. Guide RNA sequences used for CRISPR-Cas9 targeted homolous replacement approach.
(XLSX)

S6 Table List of genotyping and sequencing primers used for construct verification. Contains a list of all primers used for genotyping of genetically modified *Plasmodium knowlesi* lines.
(XLSX)
