# Supplementary figures and images for: Using Plasmodium knowlesi as a model for screening Plasmodium vivax blood-stage malaria vaccine targets reveals new candidates
Source: PLoS Pathog. 2021 Jul 1;17(7):e1008864. doi: 10.1371/journal.ppat.1008864 (PMC8279373; doi:10.1371/journal.ppat.1008864)

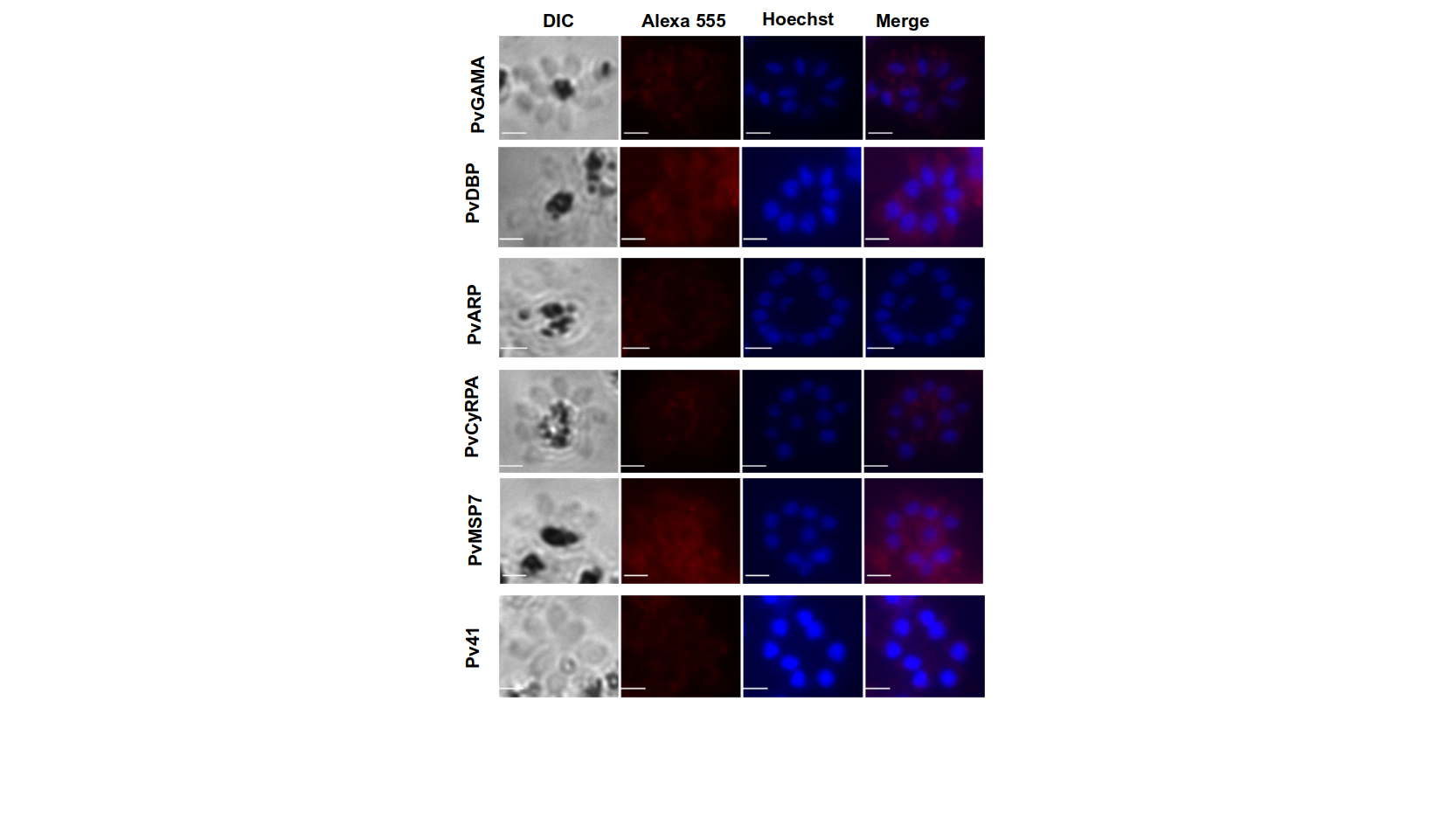

Supplement: S1 Fig — Localisation of using pre-immune serum from rabbits before they were immunized with PvGAMA, PvDBP, PvARP, PvCyRPA, PvMSP7.1 and Pv41, respectively. No specific staining is detected using Alexa 555-labeled anti-rabbit secondary antibody. Merge is an overlay of Alexa 555 and Hoeschst (parasite nuclei). Scale bar is 2 micrometers. (TIFF) [file ppat.1008864.s001.tiff]

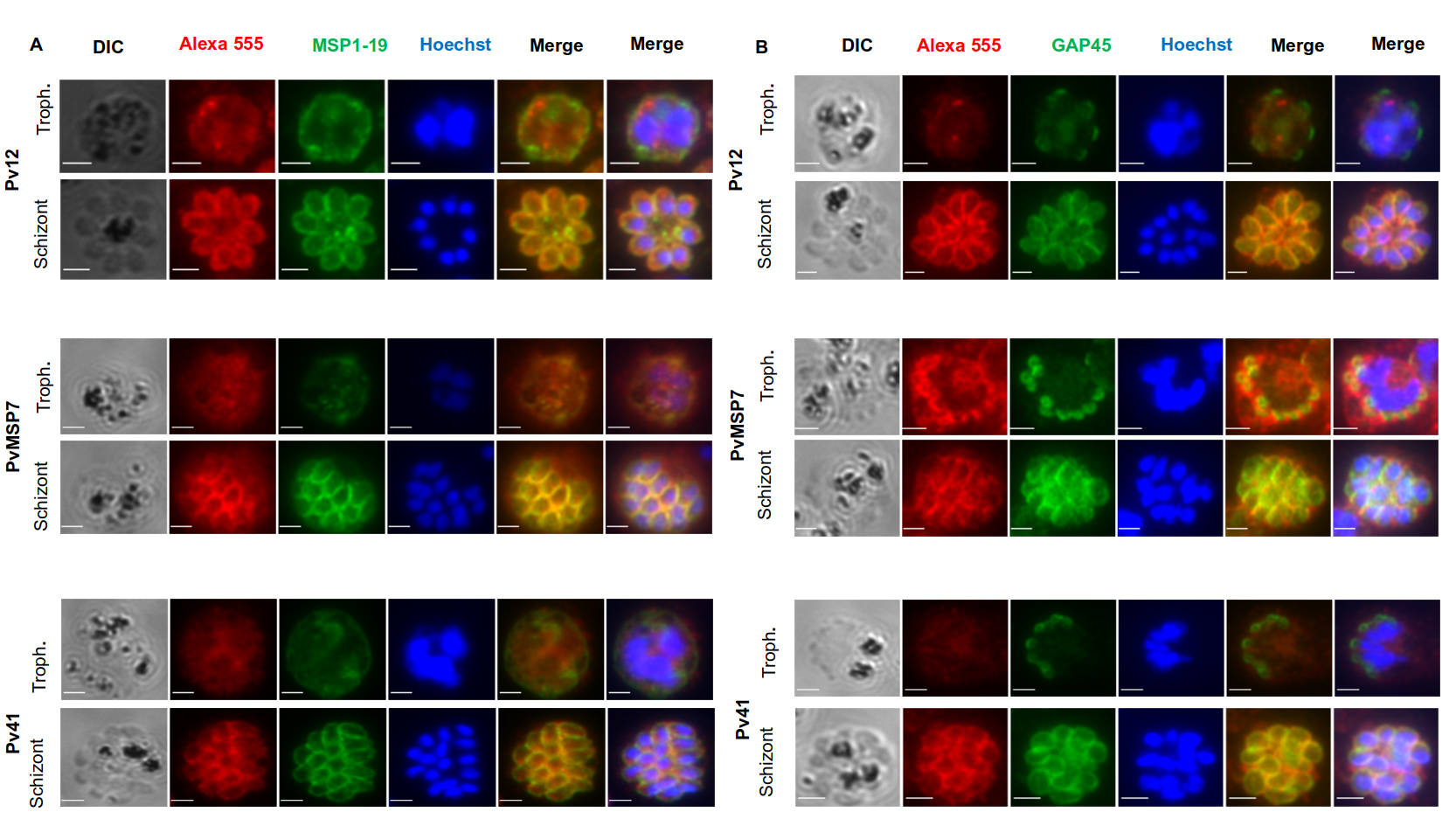

Supplement: S2 Fig — P. knowlesi proteins were localised in schizont and trophozoite (troph) stage parasites using rabbit polyclonal antibodies raised against Pv12, PvMSP7.1 and Pv41 and co-stained with rat antibodies against A) anti-PkMSP1 or B) anti-PfGAP45 for colocalization (origin of antibodies is described in Methods). Alexa Fluor 555 goat-anti rabbit and Alexa Fluor 488 goat-anti rat were used as secondary antibodies, and parasite DNA localised using Hoechst 33342. The first Merge column is one is an overlay of Alexa 555 and Alexa488 staining, while the second Merge column is an overlay of Alexa 555, Alexa488 and Hoeschst. Scale bar is 2 micrometers. (TIFF) [file ppat.1008864.s002.tiff]

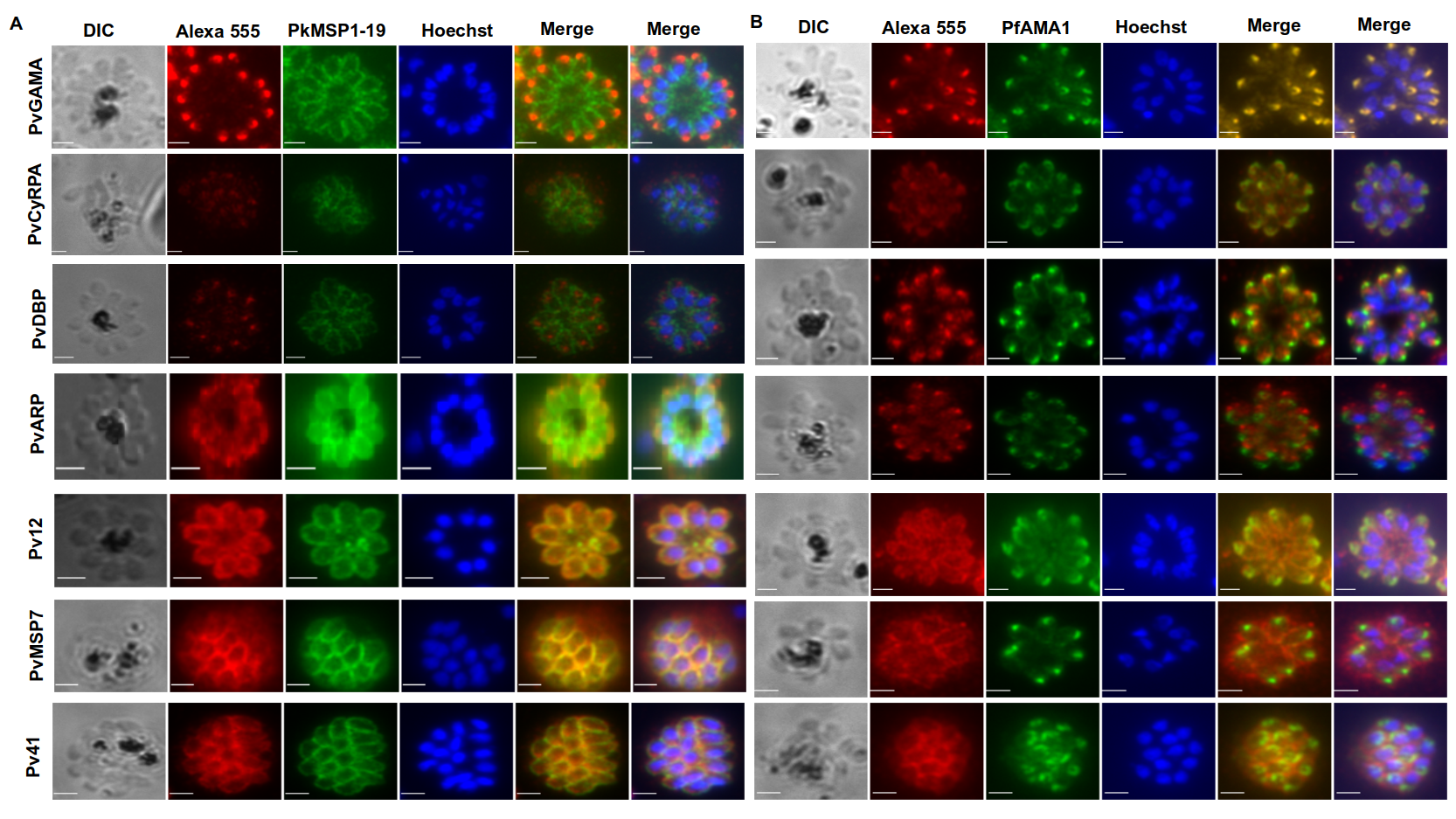

Supplement: S3 Fig — P. knowlesi proteins were localised in schizont stage parasites using rabbit polyclonal antibodies raised against P. vivax vaccine candidates and co-stained with rat antibodies against A) anti-PkMSP1 or B) anti-PfAMA1 for colocalization (origin of antibodies is described in Methods). Alexa Fluor 555 goat-anti rabbit and Alexa Fluor 488 goat-anti rat (MSP1) or mouse (AMA1) were used as secondary antibodies, and parasite DNA localised using Hoechst 33342. The first Merge column is one is an overlay of Alexa 555 and Alexa488 staining, while the second Merge column is an overlay of Alexa 555, Alexa488 and Hoeschst. Scale bar is 2 micrometers. (TIFF) [file ppat.1008864.s003.tiff]

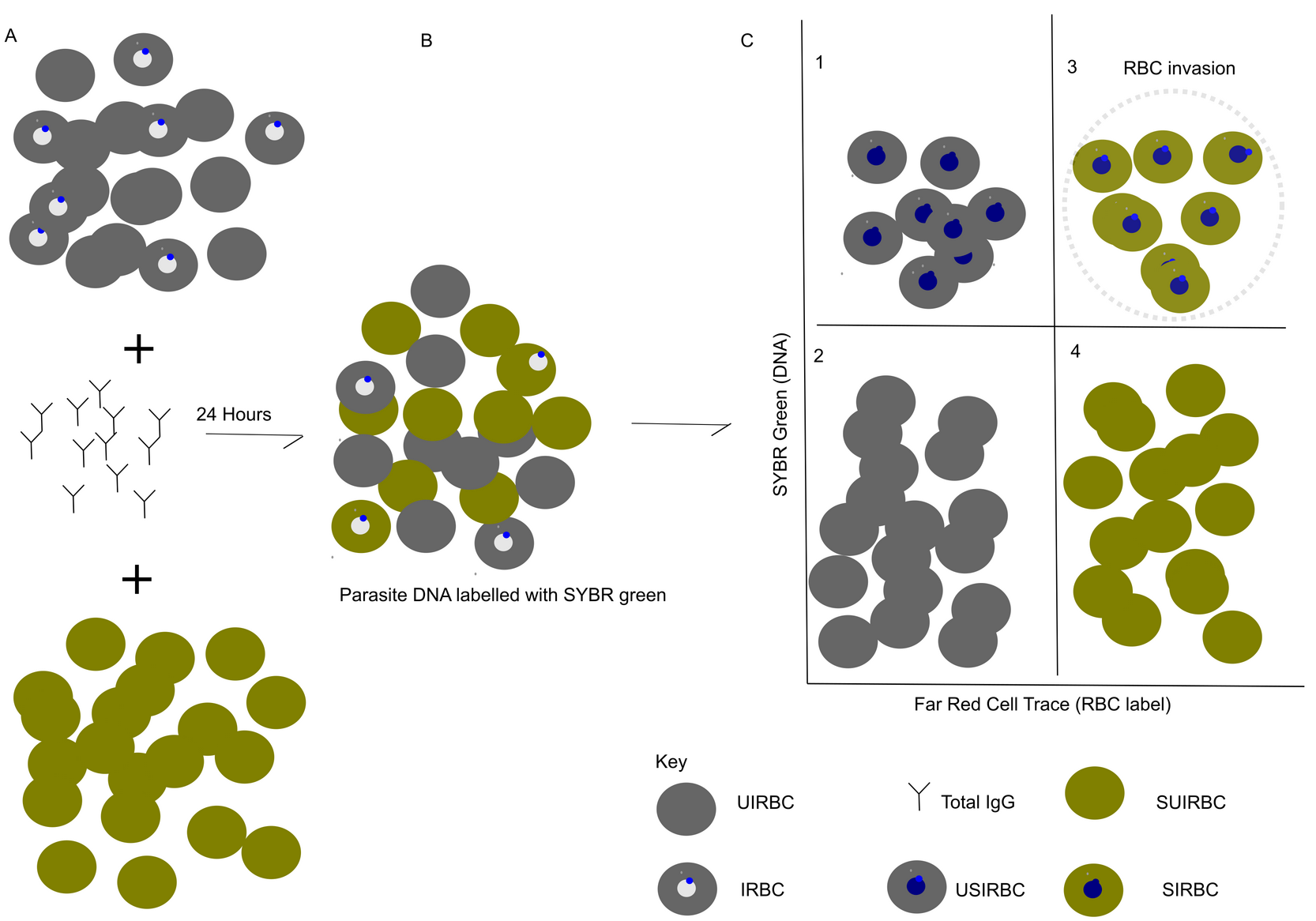

Supplement: S4 Fig — A) Synchronized P. knowlesi cultures at ring stage (IRBC) were mixed with Far-Red Cell Trace dye stained Uninfected RBCs (SUIRBC) and two-fold serial dilutions from 10 mg/ml to 0.625 mg/ml of purified total IgG. B) The mixture was incubated for 24 hours under normal culture conditions described in the Material and Methods section to allow a full cycle of development and invasion to occur. C) Parasite DNA was labelled with SYBR Green, and newly invaded parasites identified using two-colour flow cytometry, where new invasions into Far-Red Cell Trace labelled RBCs can be detected in the upper right quadrant (3—circled). (TIFF) [file ppat.1008864.s004.tiff]

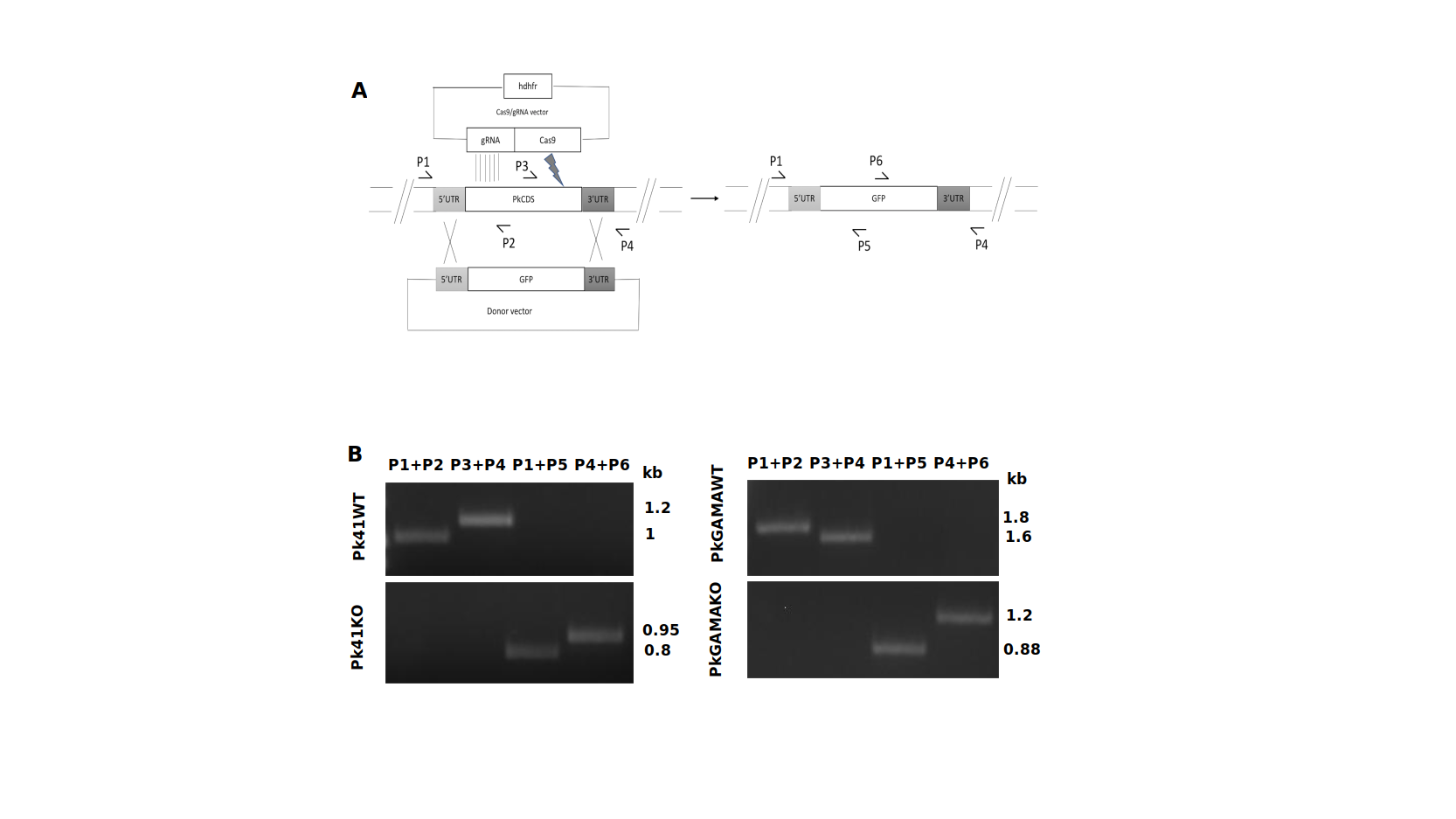

Supplement: S5 Fig — A) General strategy used to attempt to knock out Pk12, Pkarp, Pkdbpalpha, Pk41 and Pkgama. Plasmids used are Cas9/gRNA vector and donor vector containing eGFP (GFP) flanked with 5’ and 3’ untranslated region (UTR) for each respective P. knowlesi gene (PkCDS). Primer pairs used for genotyping are P1&P2 and P3&P4, to test for the presence of wildtype gene, and P1&P5 and P6&P4 to test for integration of the knockout construct. B) Genotyping of Pk41KO and PkGAMAKO with above primer pairs as compared to WT. On the right side are the obtained molecular weight in kilobase pairs (kb). (TIFF) [file ppat.1008864.s005.tiff]

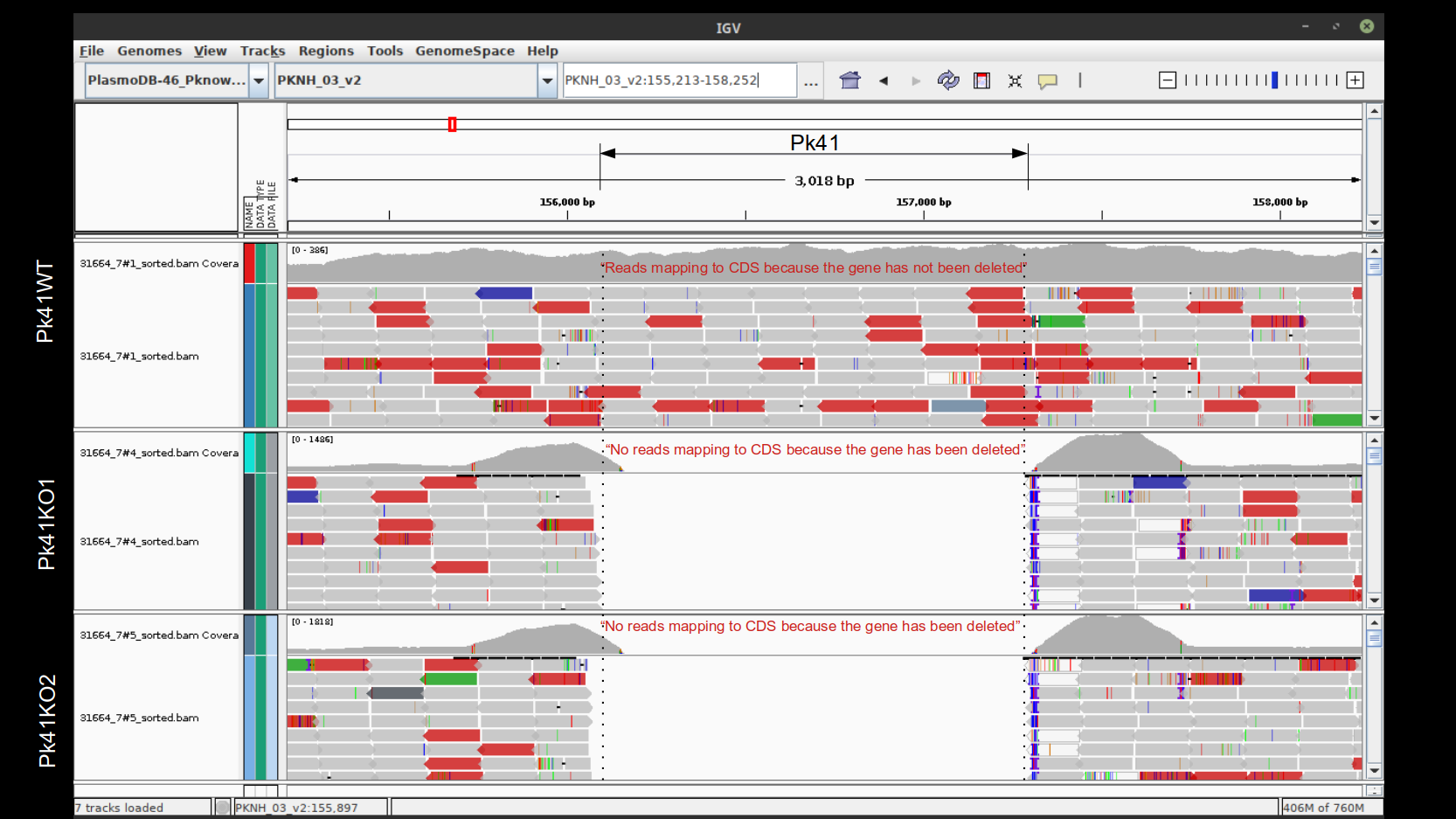

Supplement: S6 Fig — Reads generated from Illumina sequencing of Pk41 knockout strains and the WT strain from which they were generated were aligned to the P. knowlesi reference genome. Absence of reads in the centre of the gene confirms deletion of this locus from the P. knowlesi genome. (TIFF) [file ppat.1008864.s006.tiff]

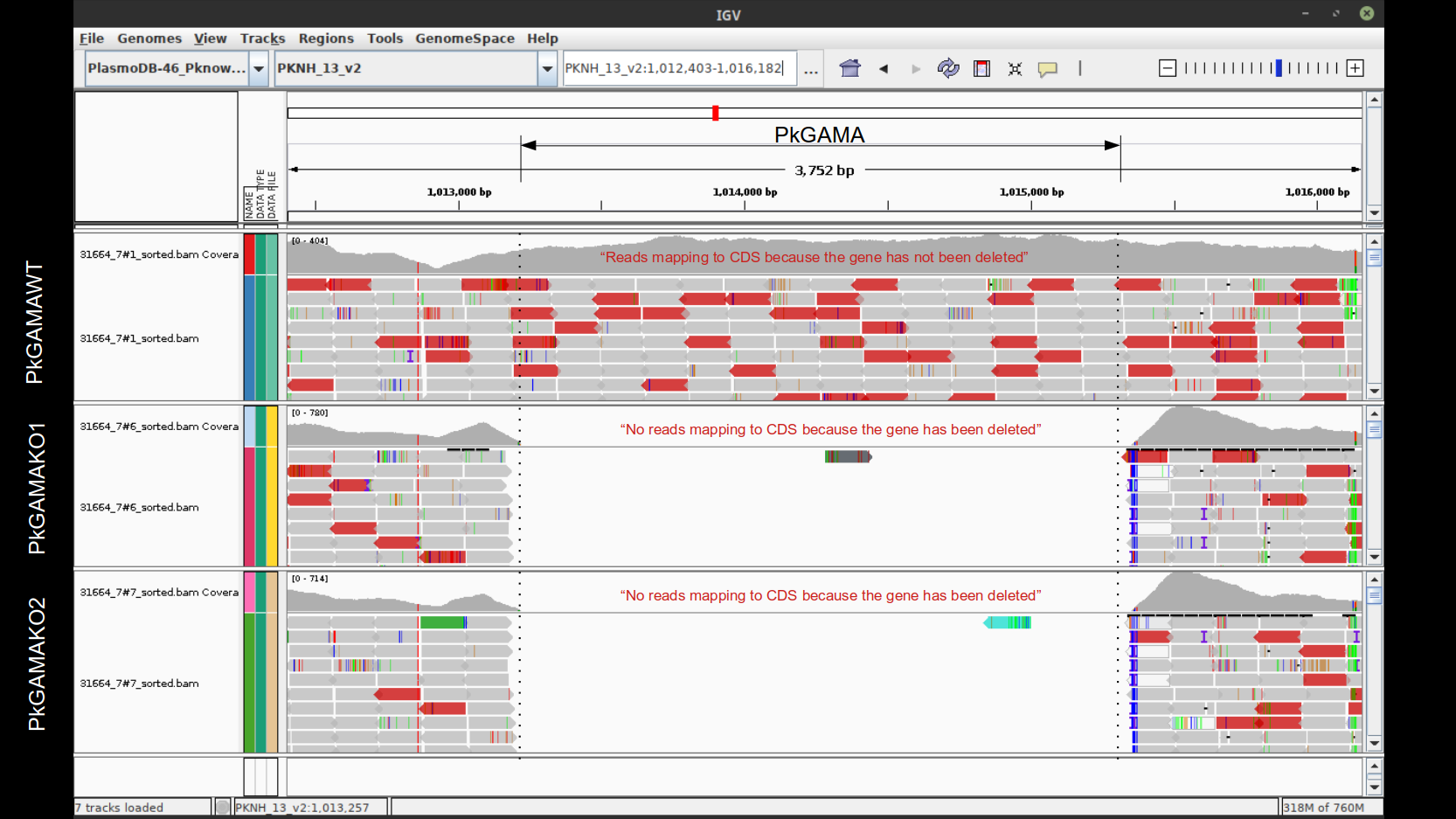

Supplement: S7 Fig — Reads generated from Illumina sequencing of PkGAMA knockout strains and the WT strain from which they were generated were aligned to the P. knowlesi reference genome. Absence of reads in the centre of the gene confirms deletion of this locus from the P. knowlesi genome. (TIFF) [file ppat.1008864.s007.tiff]

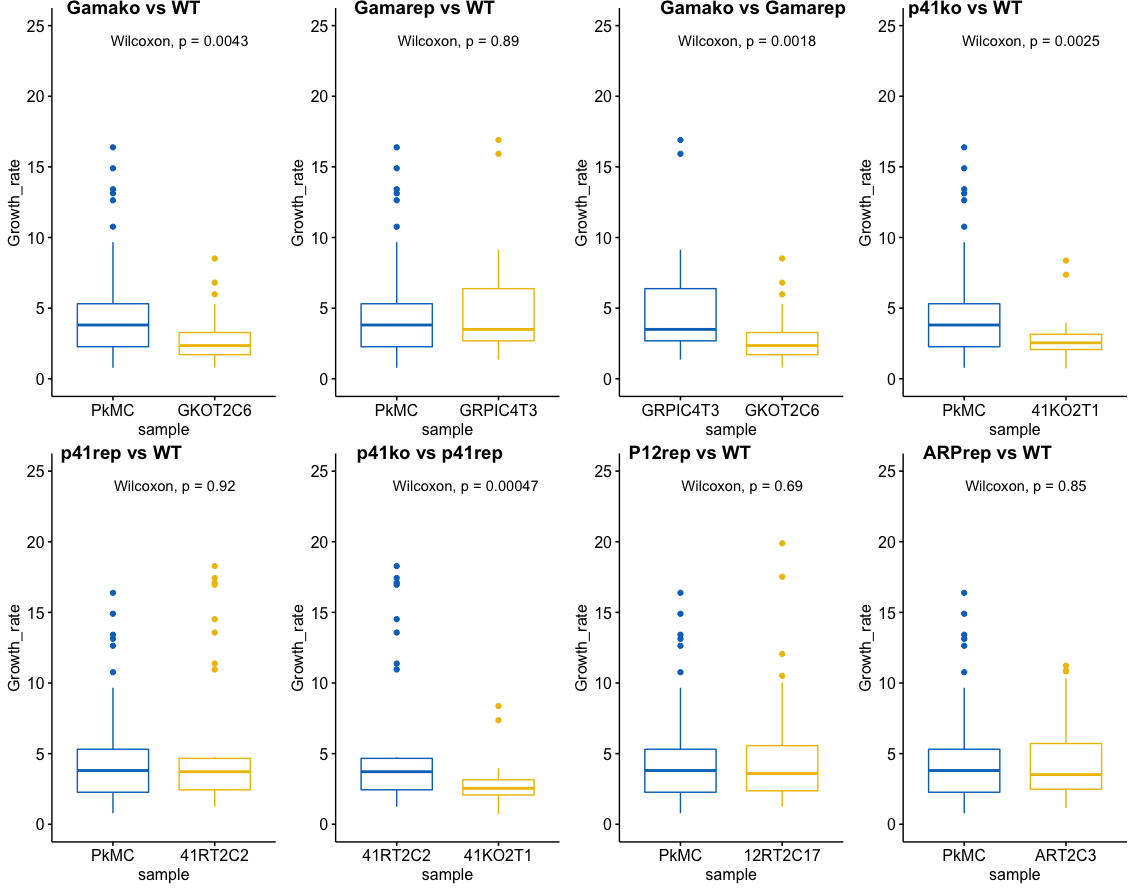

Supplement: S8 Fig — WT (P. knowlesi WT, PkMC), Gamako (PkGama knock-out clone), Gamarep (PkGama replacement clone), p41ko (Pkp41 knock out clone), p41rep (Pkp41 replacement clone), P12rep (Pkp12 replacement clone), and ARPrep (PkARP replacement clone) strains were tightly synchronised, and parasitemia measured every day using flow cytometry, then readjusted to the same parasitemia again. Parasitmia was quantified in this manner for 19 days (ie. over 19 growth cycles) and the average growth rate per cycle calculated for each strain. All strains were grown in triplicate. (TIFF) [file ppat.1008864.s008.tiff]

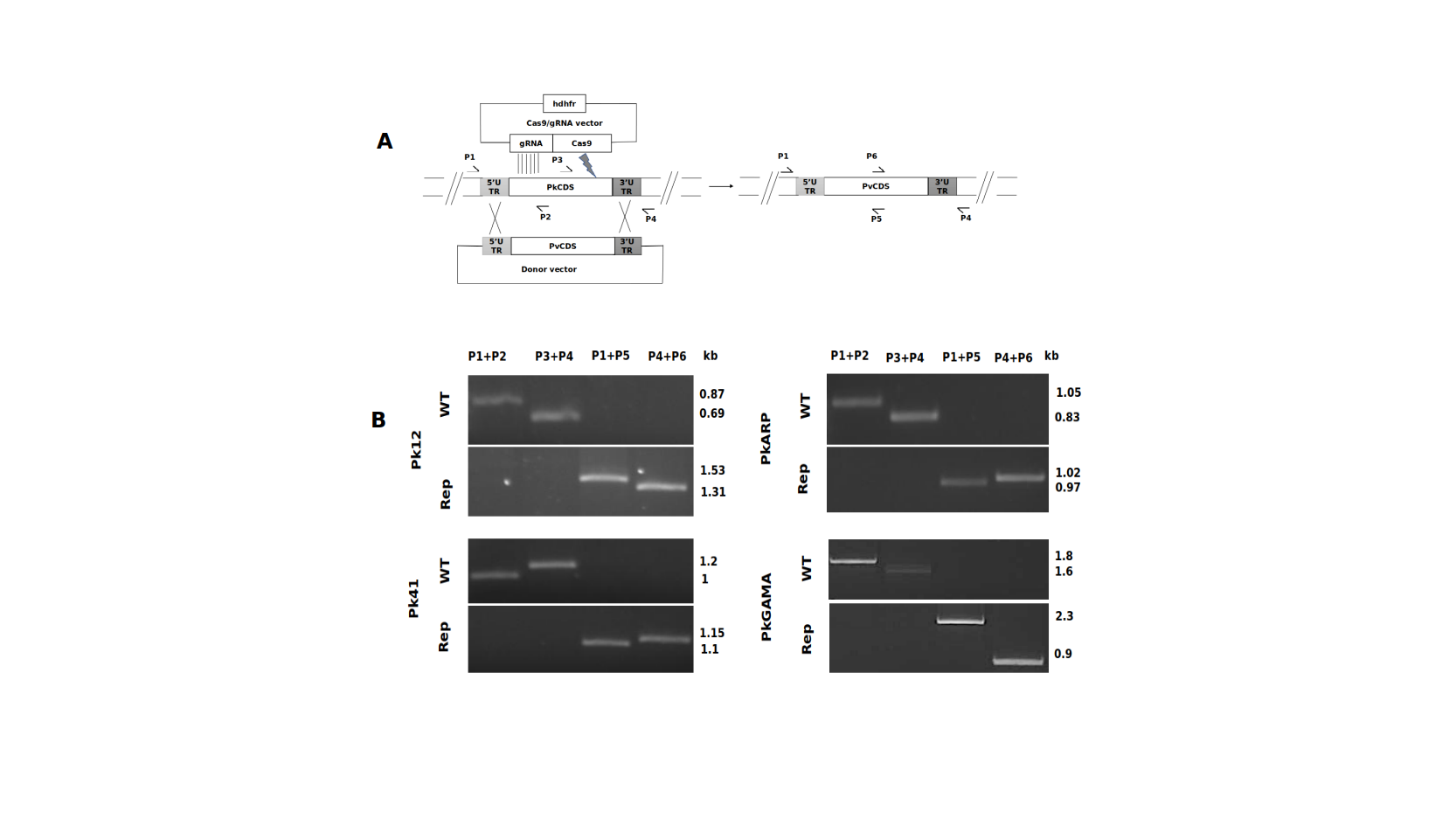

Supplement: S9 Fig — A) General strategy used to replace pk12, pkarp, pk41 and pkgama with pv12, pvarp, pv41 and pvgama, respectively. Plasmids used are Cas9/gRNA vector and donor vector containing the P. vivax coding sequence (PvCDS) flanked with 5’ and 3’ untranslated region (UTR) for each respective P. knowlesi gene (PkCDS). Primer pairs used for genotyping are P1&P2 and P3&P4, to test for the presence of the wildtype gene. P1&P5 and P6&P4 to test for integration of the replacement construct. B) Genotyping of pk12, pkarp, pk41 and pkgama allele replacement (Rep) using the above primer pairs as compared to WT. On the right side are the obtained molecular weight in kilobase pairs (kb). (TIFF) [file ppat.1008864.s009.tiff]

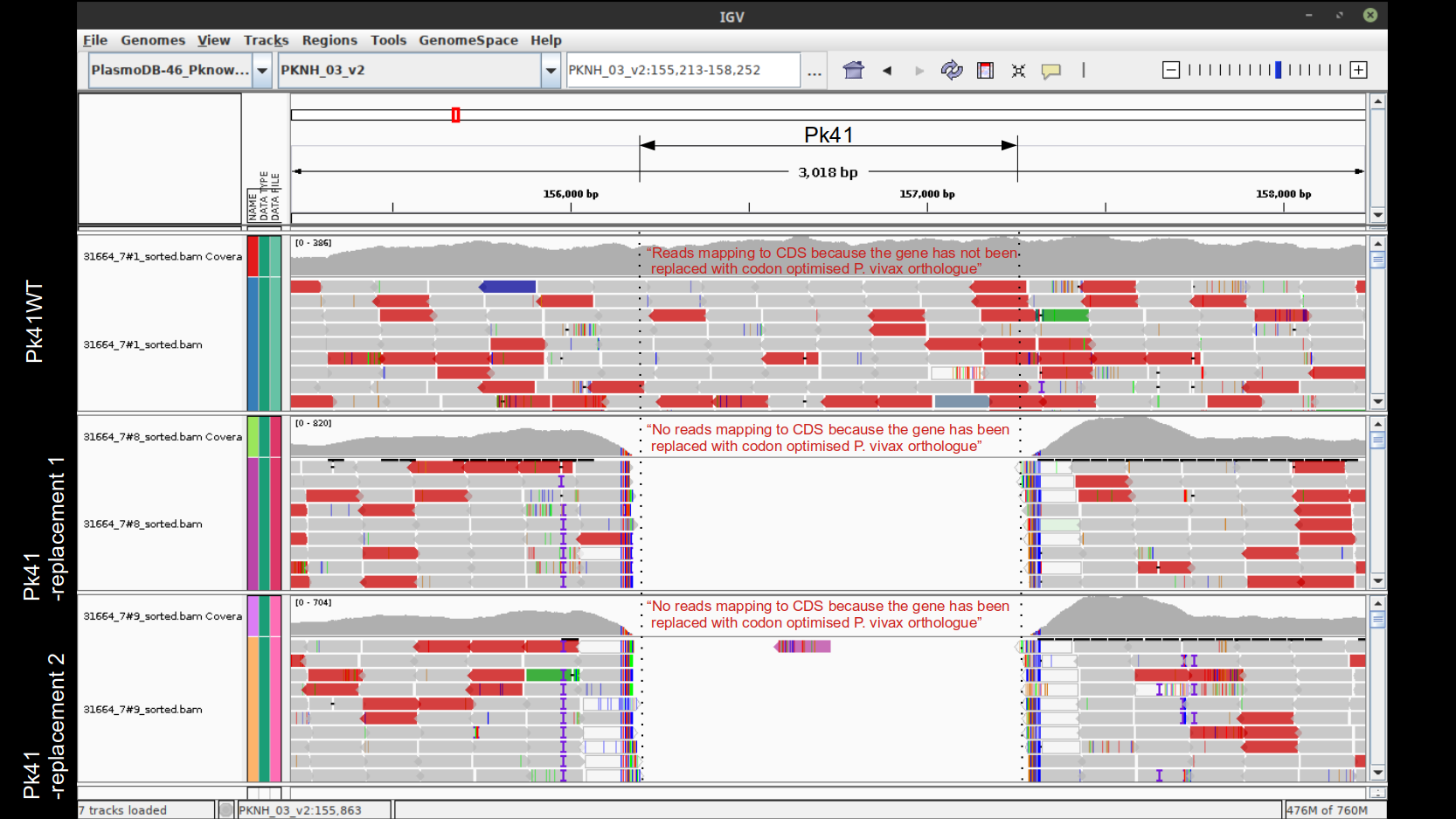

Supplement: S10 Fig — Reads generated from Illumina sequencing of PkPv41 allele replacement strains and the WT strain from which they were generated were aligned to the P. knowlesi reference genome. The P. vivax gene construct was codon optimised to human codons to reduce the chance that recombination would occur within the ORF, rather than within the flanking regions, and hence favour complete replacement of the P. knowlesi gene. As a result, reads from the replacement gene do not map to the P. knowlesi genome, confirming that the endogenous gene has been replaced. (TIFF) [file ppat.1008864.s010.tiff]

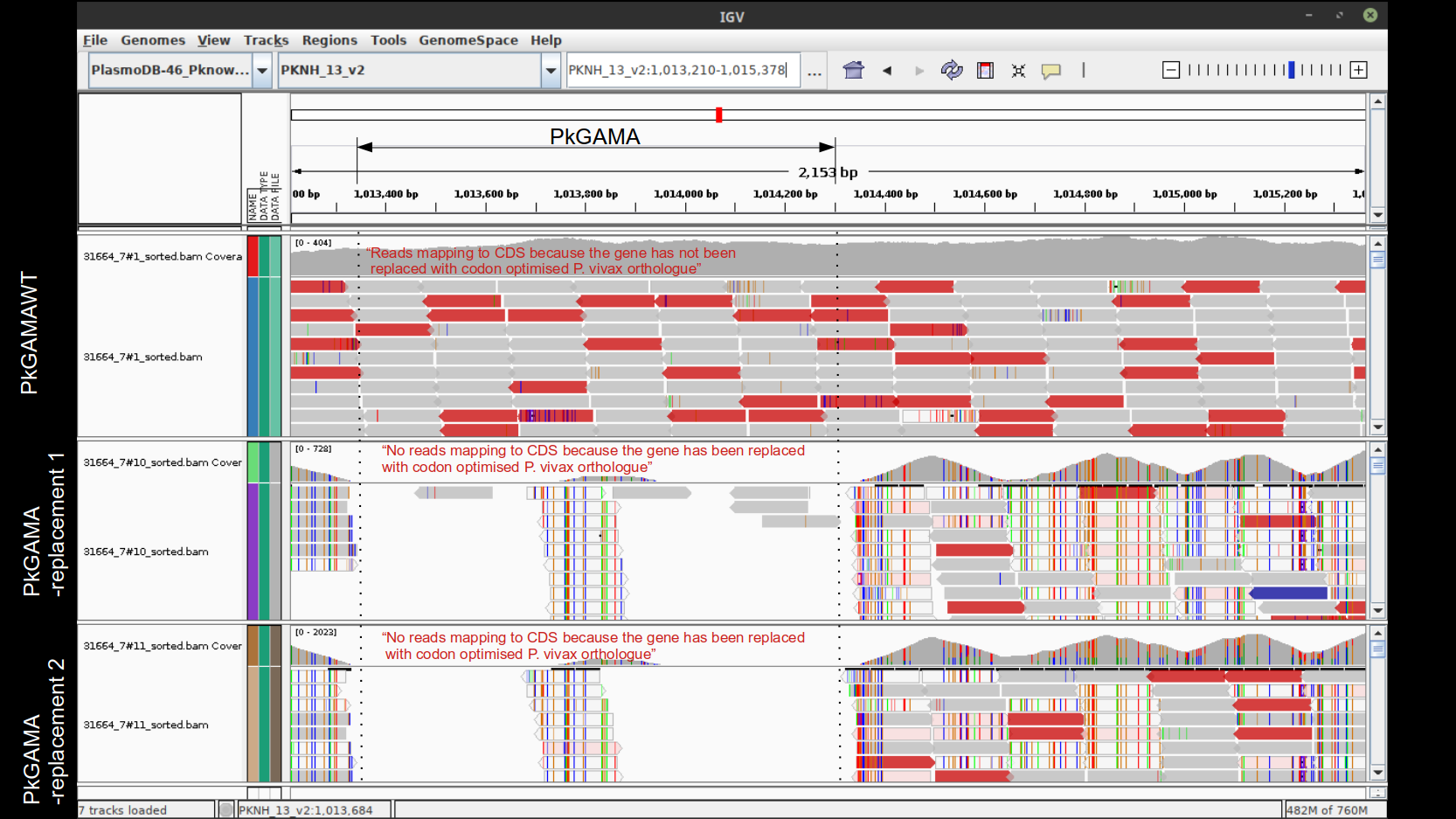

Supplement: S11 Fig — Reads generated from Illumina sequencing of PkPvGAMA allele replacement strains and the WT strain from which they were generated were aligned to the P. knowlesi reference genome. The P. vivax gene construct was codon optimised to human codons to reduce the chance that recombination would occur within the ORF, rather than within the flanking regions, and hence favour complete replacement of the P. knowlesi gene. As a result, reads from the replacement gene do not map to the P. knowlesi genome, confirming that the endogenous gene has been replaced. (TIFF) [file ppat.1008864.s011.tiff]

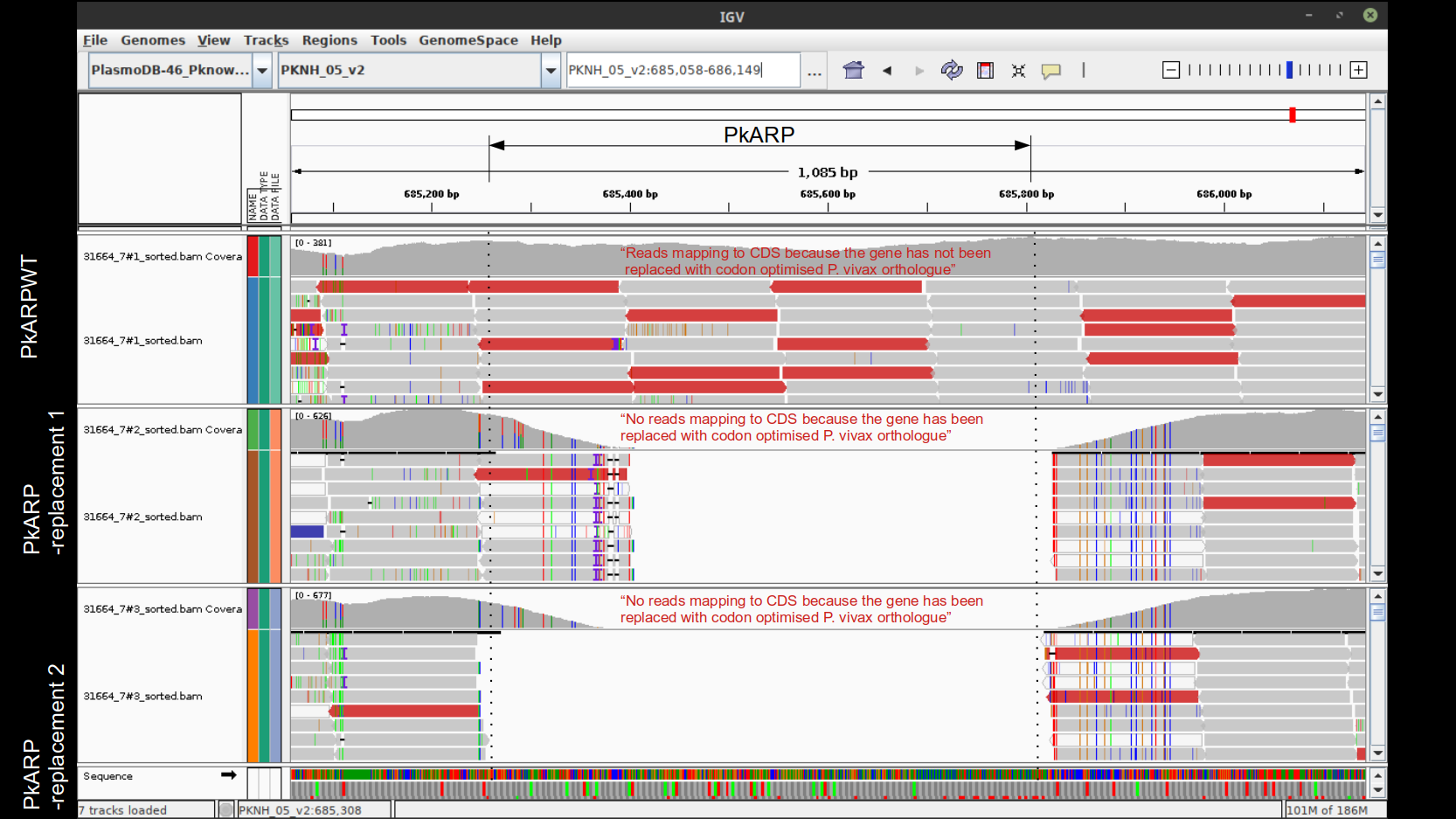

Supplement: S12 Fig — Reads generated from Illumina sequencing of PkPvARP allele replacement strains and the WT strain from which they were generated were aligned to the P. knowlesi reference genome. The P. vivax gene construct was codon optimised to human codons to reduce the chance that recombination would occur within the ORF, rather than within the flanking regions, and hence favour complete replacement of the P. knowlesi gene. As a result, reads from the replacement gene do not map to the P. knowlesi genome, confirming that the endogenous gene has been replaced. (TIFF) [file ppat.1008864.s012.tiff]

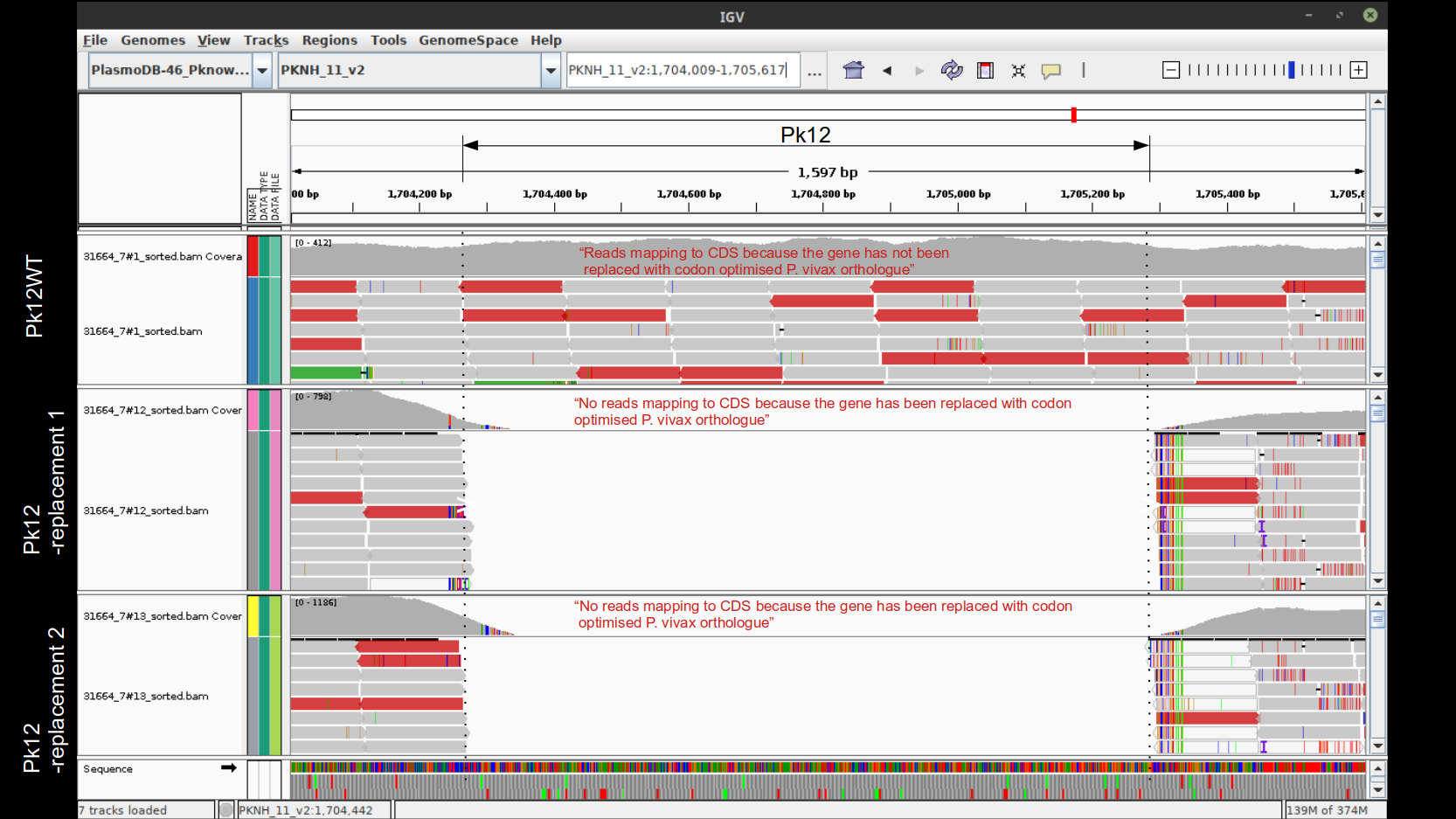

Supplement: S13 Fig — Reads generated from Illumina sequencing of PkPv12 allele replacement strains and the WT strain from which they were generated were aligned to the P. knowlesi reference genome. The P. vivax gene construct was codon optimised to human codons to reduce the chance that recombination would occur within the ORF, rather than within the flanking regions, and hence favour complete replacement of the P. knowlesi gene. As a result, reads from the replacement gene do not map to the P. knowlesi genome, confirming that the endogenous gene has been replaced. (TIFF) [file ppat.1008864.s013.tiff]

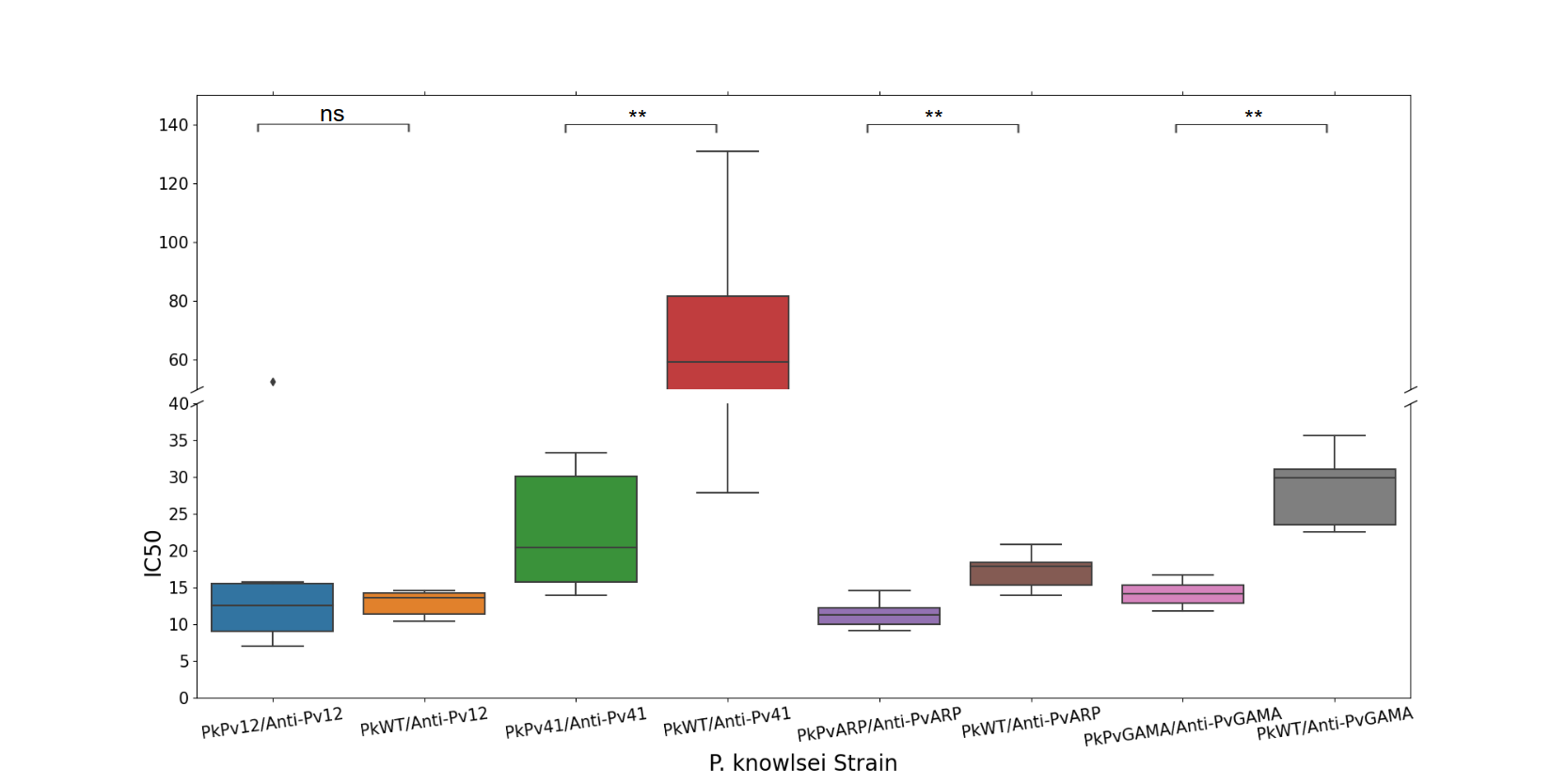

Supplement: S14 Fig — Using the invasion inhibition assay data from Fig 8 above, IC50 in mg/ml was generated using Robust linear regression model. To determine if there were statically significant differences between these values, IC50 values obtained from the allele replacement mutants (Replacements) were independently compared to P. knowlesi wild-type IC50 (PKWT) that had been treated with the same antibodies using Mann-Whitney-Wilcoxon test with a p value threshold of 5.00e-02. *: 1.00e-02 < p value < = 5.00e-02. **: 1.00e-03 < p value < = 1.00e-02. (TIFF) [file ppat.1008864.s014.tiff]

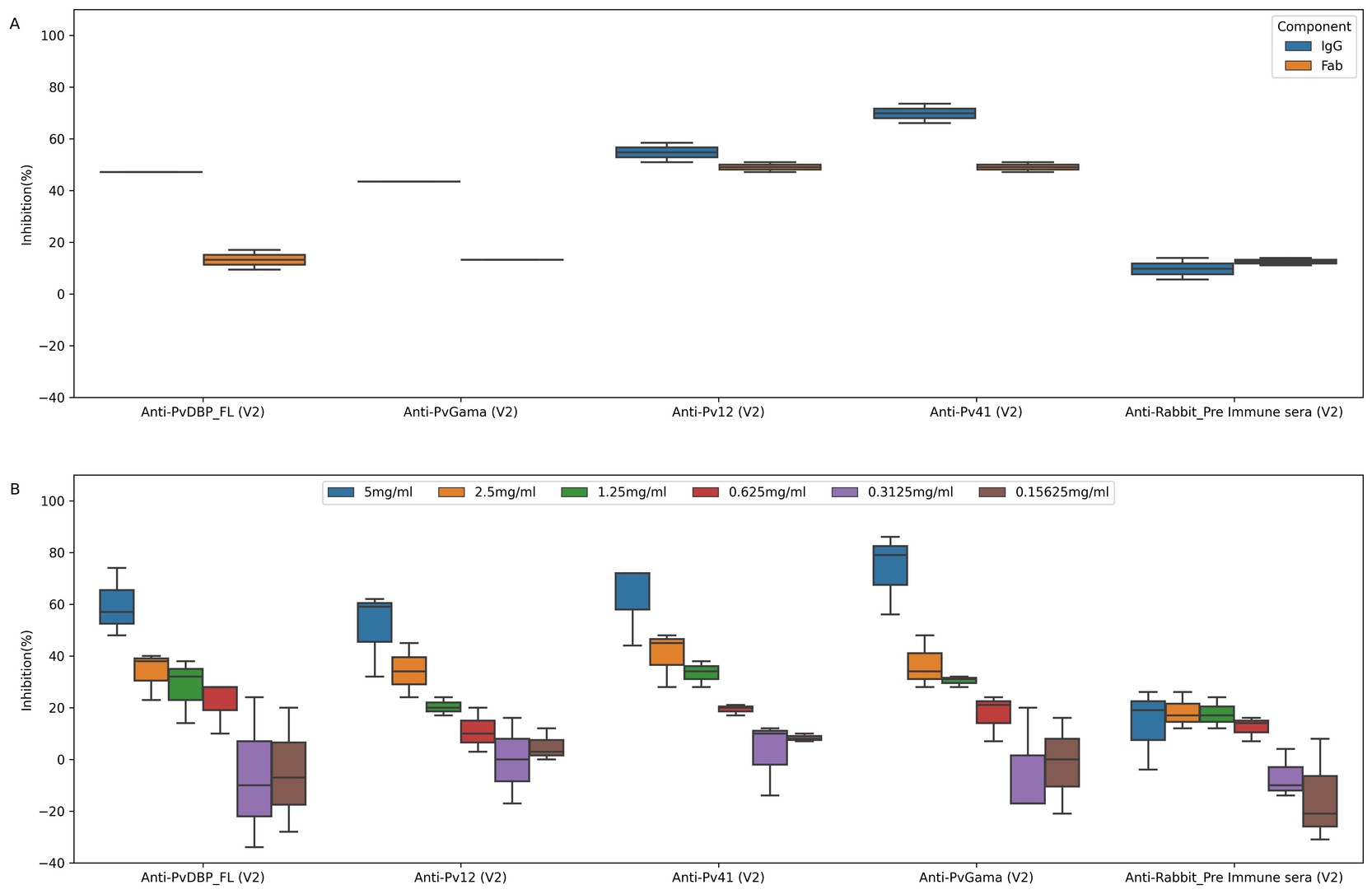

Supplement: S15 Fig — Rabbit polyclonal antibodies were raised against PvDBP, Pv12, Pv41 and PvGAMA as described in the Methods, and used in Growth Inhibition Assays as outlined in S4 Fig using either a single dose of purified Fab (A) or a dose dilution series of total IgG (B). (TIFF) [file ppat.1008864.s015.tiff]
